# Treatment Discontinuation and Adherence in Patients With Chronic Hepatitis B Infection Newly Initiating Nucleos(t)ide Analogues in Japan: A Retrospective Cohort Study

**DOI:** 10.1111/jvh.70062

**Published:** 2025-08-14

**Authors:** Shinya Kawamatsu, Kiran K. Rai, Vera Gielen, Amisha Patel, Olivia Massey, Seth W. Anderson, Yutaka Handa, Ethan Yichen Lee, Poppy Payne, Isabel Jimenez, Kejsi Begaj, Shayon Salehi, Jun Inoue, Afisi S. Ismaila

**Affiliations:** ^1^ GSK Tokyo Japan; ^2^ Adelphi Real World Bollington UK; ^3^ GSK London UK; ^4^ GSK Collegeville Pennsylvania USA; ^5^ Division of Gastroenterology Tohoku University Graduate School of Medicine Sendai Japan; ^6^ Department of Health Research Methods, Evidence and Impact McMaster University Hamilton Ontario Canada

**Keywords:** antiviral therapy, hepatitis B, nucleos(t)ide analogues, treatment adherence, treatment discontinuation

## Abstract

Nucleos(t)ide analogue (NA) therapy is the current standard of care for chronic hepatitis B (CHB) virus infection but rarely achieves functional cure, necessitating long‐term therapy, which often leads to nonadherence and increased treatment burden. This retrospective cohort study was designed to describe treatment discontinuation and adherence to second‐generation NAs among patients with CHB in Japan. We used the Japanese Medical Data Center Claims Database (JMDC Inc.) to identify adults with CHB who were newly initiated on a single‐agent, second‐generation NA between January 2007 and August 2023. Outcomes included treatment discontinuation and adherence, treatment restart after discontinuation, NA switching and factors associated with treatment discontinuation/adherence. Of the 2473 patients included in this study (mean age 49.9 years), 65.6% were male. The most common index NAs were entecavir (55.5%) and tenofovir alafenamide fumarate (TAF, 36.2%). Treatment discontinuation was observed in 20.3% of patients; mean time to discontinuation was 20.4 months. Of the patients who discontinued, 50.7% restarted NAs. Mean adherence (proportion of days covered [PDC]) was 0.87, and 81.2% of participants had PDC ≥ 80%. Age group 35–64 years, index treatment TAF and baseline hepatocellular carcinoma diagnosis were significantly associated with a decreased probability of treatment discontinuation and nonadherence. Although a high proportion of patients were persistent and adherent to NA treatment, there is a subgroup of patients whose needs are not met while receiving NA treatment, particularly in younger age groups. The results emphasise the need for alternative therapies with shorter, finite treatment durations to improve patient persistence, adherence and outcomes.

## 1 Introduction

Hepatitis B virus (HBV) infection continues to pose a global public health challenge, with 257.5 million people worldwide diagnosed in 2022, resulting in 1.1 million HBV‐related deaths [1, 2]. The Asia‐Pacific region bears the greatest global burden of HBV infection, accounting for 65% of the total cases [3]. Despite the 2010 implementation of a national action plan to combat viral hepatitis, an estimated 0.8% of Japan's population is infected with HBV [1, 4]. Persistence of hepatitis B surface antigen (HBsAg) in the serum for longer than 6 months indicates chronic infection [5]. However, not every patient with a chronic HBV infection will develop chronic hepatitis B (CHB; i.e., progress to liver disease) [6, 7]. Poor management of the infection in the long term can lead to liver disease progression and eventually to death [8]. Second‐generation nucleos(t)ide analogues (NAs)—entecavir, tenofovir disoproxil fumarate (TDF) and tenofovir alafenamide fumarate (TAF)—are the first‐line treatment for CHB in Japan because of their high genetic barrier to resistance [9]. Functional cure, defined as sustained HBsAg loss and HBV DNA less than the lower limit of quantification at 24 weeks off therapy, rarely happens with NA therapy (< 5%) [10, 11, 12]. The Japan Society of Hepatology guidelines define HBsAg elimination as the long‐term goal of antiviral therapy for patients with persistent HBV infection [9]. The three short‐term goals of antiviral treatment before elimination of HBsAg are persistent normalisation of alanine transaminase, achievement of negative hepatitis B e (HBe) antigen and positive anti‐HBe antibody status, and suppression of HBV DNA replication [9].

NAs inhibit viral polymerase activity in the infected hepatocytes, resulting in decreased production of virions, but do not inhibit the de novo formation of viral covalently closed circular DNA (cccDNA) in newly infected cells [13]. Decrease in cccDNA with NA treatment occurs only during long‐term therapy [13], which is often associated with nonadherence, adverse events in patients and an increased burden to the healthcare system [14, 15, 16]. Studies have linked poor adherence to antiviral therapy with an increased risk of virological breakthrough [17, 18, 19, 20], and NA resistance [17, 21].

Adherence to and persistence with NA therapies is vital to achieve sustained virological suppression and to prevent disease progression. However, as is common with long‐term therapies for chronic conditions, maintaining sustained adherence to NA therapy can be challenging [22]. Forgetfulness, a limited understanding of adherence and disruptions to established routines have been cited as barriers to treatment adherence [15]. These difficulties often lead to suboptimal adherence, thereby compromising treatment efficacy [22], and increase the risk of treatment discontinuation, which may lead to clinical or virological relapse [23]. Current evidence on these outcomes in Japan is limited to a single‐site study of small cohort size [19]. A more comprehensive understanding of the longitudinal treatment patterns at a population level is key to contextualise discontinuation and adherence and to describe the real‐world utilisation of these therapies. This study aimed to assess the discontinuation and adherence of newly initiated single‐agent, second‐generation NAs in the treatment of patients with CHB in Japan.

## 2 Methods

### 2.1 Study Design and Population

This was a retrospective longitudinal cohort study using anonymized claims data from the Japan Medical Data Center (JMDC Inc.), which includes data on ~17 million insured persons from health insurance providers for company employees and their dependents. The study period spanned January 1, 2006–December 31, 2023, and the indexing period was between January 1, 2007, and August 31, 2023. Baseline was defined as the 12‐month period prior to and excluding the index date, and the follow‐up period was defined as a minimum of 4 months following and including the index date. Patients were censored at the earliest of the following: coinfection, end of continuous enrollment, patient death or end of study period (Figure S1). Patients with CHB newly initiating a single‐agent, second‐generation NA treatment (entecavir, TDF or TAF) were indexed on the date of the first recorded claim for an NA treatment during the indexing period.

Included patients were NA‐naïve adults (aged ≥ 18 years) with at least one pharmacy claim (inpatient or outpatient) for a single‐agent, second‐generation NA during the indexing period, at least one claim with a diagnosis code for CHB virus infection (inpatient or outpatient) during the baseline period, and at least 1 year and at least 4 months of continuous enrolment in claims data during the baseline and follow‐up periods, respectively. Patients with at least one claim with a diagnosis of coinfections (hepatitis C, hepatitis D or human immunodeficiency virus), those with at least one claim for a first‐ (lamivudine and adefovir dipivoxil) or second‐generation (entecavir, telbivudine, TDF and TAF) NA, and those with two or more claims for different second‐generation NA treatments during the baseline period were excluded.

### 2.2 Study Outcomes

#### 2.2.1 Demographic and Clinical Characteristics

Baseline demographic characteristics included age at index, sex, index year and length of follow‐up. Clinical characteristics included Quan‐Charlson Comorbidity Index (CCI) and CHB‐related comorbidities (i.e., cirrhosis [compensated and decompensated], hepatocellular carcinoma (HCC), liver failure, nonalcoholic fatty liver disease and liver transplant).

#### 2.2.2 NA Treatment Discontinuation

Discontinuation was defined as a continuous treatment gap of ≥ 90 days without drug supply; the date of discontinuation was defined as the last day's supply of NA treatment before a gap of 90 days or more in treatment. Median time to discontinuation (TTD) was defined as the time from newly initiated NA treatment date to the last day of treatment before discontinuation was reported. Medication switching from one second‐generation NA to another was not considered as discontinuation.

#### 2.2.3 NA Treatment Restart

Treatment restart was defined as patients restarting second‐generation NA treatment after discontinuation (i.e., 90 days after discontinuation date).

#### 2.2.4 NA Treatment Adherence

Adherence was considered as the proportion of days covered (PDC) ≥ 80% from the index date to the end of follow‐up. Medication switching from one second‐generation NA to another was allowed and counted towards the total days covered.

#### 2.2.5 NA Treatment Switching

NA treatment switches from the index NA treatment to another second‐generation single‐drug formulation were assessed. Switching did not require discontinuation and could occur at any time after the index date if a claim for a different single‐agent, second‐generation NA occurred, even if it overlapped with an existing NA treatment. Patients with multiple switches were only counted once when reporting the proportion of patients switching NA treatment. However, when a patient switched treatment multiple times, all switches were included when reporting the number of switches.

#### 2.2.6 Factors Associated With Treatment Discontinuation and Adherence

Factors associated with treatment discontinuation were assessed, with the outcome defined as time to indexed NA treatment discontinuation. Factors associated with adherence to NA treatment were assessed, where PDC ≥ 0.80 was used as the definition for adherence and the outcome of interest. Patient demographic (i.e., age at index, sex and length of follow‐up) and clinical characteristics (i.e., Quan‐CCI, CHB‐related comorbidities of interest) and indexed NA treatment were covariates of interest.

#### 2.2.7 Possible Reasons for Discontinuing Index NA Treatment

Assessed as the presence (physician‐guided) or absence (non‐physician‐guided) of three or more laboratory test orders/claims for liver function, HBV DNA or HBV antigen (HBsAg and hepatitis core‐related antigen) tests within 90 days pre‐ and post‐discontinuation date.

All outcomes detailed above, except factors associated with treatment discontinuation and adherence, were stratified by age (in years) at index and by index NA treatment.

### 2.3 Data Analysis

The proportions of patients who discontinued and restarted NA treatment were reported descriptively during variable follow‐up and at fixed time intervals (12, 24, 36, 48 and 60 months). The median time from each discontinuation to restarting treatment, along with respective time‐to‐event analyses (i.e., the probability of not discontinuing treatment and the probability of restarting), was calculated. Rates of treatment discontinuation, restart and switching, per 100 person‐years, were also calculated by dividing the total number of events by the total time at risk summed across all patients.

PDC was reported descriptively during variable follow‐up and at fixed time intervals and was calculated by dividing the total number of days' medication prescribed before discontinuation (i.e., total days covered) by the total number of days of follow‐up.

A sensitivity analysis, where discontinuation was defined as a treatment gap of ≥ 60 days, was performed to consider the effects of treatment discontinuation. A further sensitivity analysis was performed to consider NA treatment adherence and discontinuation prior to (January 1, 2007–April 6, 2020), during (April 7, 2020–September 30, 2021) and post (October 1, 2021–August 31, 2023) the COVID‐19 pandemic [24]. The results of the sensitivity analyses are reported in the Supporting Information.

A Cox proportional hazard model was used to identify factors associated with NA treatment discontinuation. A logistic regression model was used to identify factors associated with NA treatment adherence. Goodness‐of‐fit was evaluated using accepted model fit statistics to determine which covariates to keep in the final model.

For descriptive analyses, numeric variables were reported as counts, mean, median, standard deviation (SD), 25th (Q1) and 75th (Q3) percentiles, and minimum and maximum values, while categorical variables were reported as relative frequencies and proportions/percentages. Time‐to‐event variables, such as number, median and associated 95% confidence intervals (CIs), were calculated using the Kaplan–Meier estimation. Cox proportional hazards and logistic regression models were reported as coefficients and odds/hazard ratios along with standard errors, z‐scores, *p*‐values and 95% CIs.

## 3 Results

### 3.1 Baseline Characteristics

The mean (SD) age of the study population (*n* = 2473; Figure S2) was 49.9 (10.4) years; 65.6% (*n* = 1623) were male and there was a numerically higher proportion of males in the 18–34 years age group (77.6%) compared with other age groups (Table 1). The mean (SD) length of follow‐up for the overall population was 51.3 (39.5) months. Patients indexed on TAF had a numerically lower mean (SD) length of follow‐up (32.2 [19.2] months) compared with patients indexed on entecavir and TDF (62.0 [45.4] and 63.4 [30.1] months, respectively).

**TABLE 1:** Baseline patient characteristics, overall and stratified by age and index NA.

	Overall (*n* = 2473)	Age at index (in years)				Index NA		
		18–34 (*n* = 170)	35–49 (*n* = 1058)	50–64 (*n* = 1039)	65+ (*n* = 206)	Entecavir (*n* = 1373)	TAF (*n* = 895)	TDF (*n* = 205)
Index year, *n* (%)								
2007	< 11	< 11	< 11	0 (0.0)	0 (0.0)	< 11	0 (0.0)	0 (0.0)
2008	< 11	0 (0.0)	< 11	< 11	0 (0.0)	< 11	0 (0.0)	0 (0.0)
2009	12 (0.5)	< 11	< 11	< 11	< 11	12 (0.9)	0 (0.0)	0 (0.0)
2010	25 (1.0)	< 11	14 (1.3)	< 11	< 11	25 (1.8)	0 (0.0)	0 (0.0)
2011	34 (1.4)	< 11	16 (1.5)	14 (1.3)	< 11	34 (2.5)	0 (0.0)	0 (0.0)
2012	155 (6.3)	< 11	77 (7.3)	65 (6.3)	< 11	155 (11.3)	0 (0.0)	0 (0.0)
2013	72 (2.9)	11 (6.5)	38 (3.6)	21 (2.0)	< 11	72 (5.2)	0 (0.0)	0 (0.0)
2014	131 (5.3)	14 (8.2)	56 (5.3)	52 (5.0)	< 11	120 (8.7)	0 (0.0)	11 (5.4)
2015	129 (5.2)	< 11	66 (6.2)	48 (4.6)	< 11	105 (7.6)	0 (0.0)	24 (11.7)
2016	176 (7.1)	< 11	76 (7.2)	74 (7.1)	17 (8.3)	117 (8.5)	0 (0.0)	59 (28.8)
2017	189 (7.6)	< 11	89 (8.4)	76 (7.3)	15 (7.3)	122 (8.9)	< 11	NR
2018	263 (10.6)	21 (12.4)	116 (11.0)	109 (10.5)	17 (8.3)	119 (8.7)	122 (13.6)	22 (10.7)
2019	294 (11.9)	21 (12.4)	123 (11.6)	128 (12.3)	22 (10.7)	115 (8.4)	165 (18.4)	14 (6.8)
2020	278 (11.2)	20 (11.8)	118 (11.2)	113 (10.9)	27 (13.1)	NR	162 (18.1)	< 11
2021	290 (11.7)	18 (10.6)	123 (11.6)	119 (11.5)	30 (14.6)	NR	178 (19.9)	< 11
2022	257 (10.4)	16 (9.4)	79 (7.5)	124 (11.9)	38 (18.4)	NR	158 (17.7)	< 11
2023	157 (6.3)	< 11	56 (5.3)	80 (7.7)	NR	54 (3.9)	NR	< 11
Age at index (in years)								
Mean (SD)	49.9 (10.4)	30.0 (3.9)	43.2 (4.1)	56.3 (4.2)	68.7 (2.9)	50.9 (10.2)	49.4 (10.5)	45.0 (10.6)
Median (IQR)	50.0 (43.0–57.0)	31.0 (28.0–33.0)	43.0 (40.0–47.0)	56.0 (53.0–60.0)	68.0 (66.0–71.0)	51.0 (44.0–58.0)	49.0 (42.0–57.0)	44.0 (36.0–53.0)
Categorised, *n* (%)								
18–34 years	170 (6.9)	170 (100.0)	0 (0.0)	0 (0.0)	0 (0.0)	78 (5.7)	NR	NR
35–49 years	1058 (42.8)	0 (0.0)	1058 (100.0)	0 (0.0)	0 (0.0)	550 (40.1)	398 (44.5)	110 (53.7)
50–64 years	1039 (42.0)	0 (0.0)	0 (0.0)	1039 (100.0)	0 (0.0)	623 (45.4)	361 (40.3)	55 (26.8)
65+ years	206 (8.3)	0 (0.0)	0 (0.0)	0 (0.0)	206 (100.0)	122 (8.9)	NR	< 11
Patient sex, *n* (%)								
Male	1623 (65.6)	132 (77.6)	715 (67.6)	659 (63.4)	117 (56.8)	945 (68.8)	553 (61.8)	125 (61.0)
Female	850 (34.4)	38 (22.4)	343 (32.4)	380 (36.6)	89 (43.2)	428 (31.2)	342 (38.2)	80 (39.0)
Length of follow‐up (in months)								
Mean (SD)	51.3 (39.5)	55.1 (44.1)	62.4 (42.5)	44.0 (34.1)	28.0 (23.1)	62.0 (45.4)	32.2 (19.2)	63.4 (30.1)
Median (IQR)	40.4 (20.4–71.8)	40.5 (20.5–79.9)	52.2 (28.7–91.1)	35.1 (16.6–61.9)	21.1 (12.7–37.4)	50.5 (23.1–98.3)	29.5 (15.6–47.7)	72.1 (37.1–88.0)
Quan‐CCI (excluding those with mild liver disease)								
Mean (SD)	1.1 (2.0)	0.3 (0.8)	0.6 (1.3)	1.5 (2.2)	2.7 (3.1)	1.3 (2.2)	0.9 (1.8)	0.7 (1.3)
Median (IQR)	0.0 (0.0–2.0)	0.0 (0.0–0.0)	0.0 (0.0–1.0)	1.0 (0.0–2.0)	2.0 (0.0–3.0)	0.0 (0.0–2.0)	0.0 (0.0–1.0)	0.0 (0.0–1.0)
Categorised, *n* (%)								
0	1500 (60.7)	139 (81.8)	786 (74.3)	514 (49.5)	61 (29.6)	760 (55.4)	598 (66.8)	142 (69.3)
1	276 (11.2)	17 (10.0)	107 (10.1)	131 (12.6)	21 (10.2)	152 (11.1)	99 (11.1)	25 (12.2)
2+	697 (28.2)	14 (8.2)	165 (15.6)	394 (37.9)	124 (60.2)	461 (33.6)	198 (22.1)	38 (18.5)
Quan‐CCI at disease class level, *n* (%)								
Mild liver disease	2446 (98.9)	168 (98.8)	1050 (99.2)	1026 (98.7)	202 (98.1)	1360 (99.1)	883 (98.7)	203 (99.0)
Malignancy	500 (20.2)	< 11	100 (9.5)	297 (28.6)	NR	333 (24.3)	143 (16.0)	24 (11.7)
Chronic pulmonary disease	254 (10.3)	18 (10.6)	92 (8.7)	113 (10.9)	31 (15.0)	147 (10.7)	84 (9.4)	23 (11.2)
Congestive heart failure	117 (4.7)	< 11	NR	69 (6.6)	25 (12.1)	74 (5.4)	31 (3.5)	12 (5.9)
Rheumatic disease	106 (4.3)	< 11	NR	59 (5.7)	21 (10.2)	72 (5.2)	NR	< 11
Metastatic solid tumour	98 (4.0)	0 (0.0)	17 (1.6)	58 (5.6)	23 (11.2)	67 (4.9)	NR	< 11
Diabetes (mild–moderate)	89 (3.6)	0 (0.0)	23 (2.2)	45 (4.3)	21 (10.2)	53 (3.9)	NR	< 11
Cerebrovascular disease	83 (3.4)	0 (0.0)	18 (1.7)	33 (3.2)	32 (15.5)	49 (3.6)	NR	< 11
Diabetes with complications	76 (3.1)	< 11	NR	44 (4.2)	16 (7.8)	55 (4.0)	NR	< 11
Severe liver disease	70 (2.8)	< 11	17 (1.6)	45 (4.3)	< 11	53 (3.9)	NR	< 11
Peripheral vascular disease	68 (2.7)	0 (0.0)	16 (1.5)	33 (3.2)	19 (9.2)	45 (3.3)	NR	< 11
Renal disease	43 (1.7)	< 11	17 (1.6)	16 (1.5)	< 11	29 (2.1)	< 11	< 11
Myocardial infarction	21 (0.8)	0 (0.0)	< 11	11 (1.1)	< 11	13 (0.9)	< 11	< 11
Dementia	< 11	0 (0.0)	0 (0.0)	< 11	< 11	< 11	< 11	0 (0.0)
Hemiplegia or paraplegia	< 11	0 (0.0)	< 11	< 11	< 11	< 11	< 11	0 (0.0)
CHB infection‐related comorbidities, *n* (%)								
NAFLD	227 (9.2)	18 (10.6)	89 (8.4)	99 (9.5)	21 (10.2)	118 (8.6)	89 (9.9)	20 (9.8)
Hepatocellular carcinoma	190 (7.7)	< 11	49 (4.6)	114 (11.0)	NR	118 (8.6)	59 (6.6)	13 (6.3)
Cirrhosis (de‐ and compensated)	153 (6.2)	11 (6.5)	52 (4.9)	73 (7.0)	17 (8.3)	98 (7.1)	NR	< 11
Liver failure	27 (1.1)	< 11	< 11	15 (1.4)	< 11	21 (1.5)	< 11	< 11
Liver transplant	< 11	0 (0.0)	0 (0.0)	< 11	0 (0.0)	< 11	0 (0.0)	0 (0.0)
Index NA, *n* (%)								
Entecavir	1373 (55.5)	78 (45.9)	550 (52.0)	623 (60.0)	122 (59.2)	1373 (100)	0 (0.0)	0 (0.0)
TAF	895 (36.2)	NR	398 (37.6)	361 (34.7)	NR	0 (0.0)	895 (100)	0 (0.0)
TDF	205 (8.3)	NR	110 (10.4)	55 (5.3)	< 11	0 (0.0)	0 (0.0)	205 (100)

*Note:* Primary data suppression applied to all values with fewer than 11 patients (< 11), with a secondary suppression (NR) applied where required to protect primary suppression.

Abbreviations: CCI, Charlson Comorbidity Index; IQR, interquartile range; NA, nucleos(t)ide analogue; NAFLD, nonalcoholic fatty liver disease; NR, not reported; SD, standard deviation; TAF, tenofovir alafenamide fumarate; TDF, tenofovir disoproxil fumarate.

Mean (SD) Quan‐CCI score of the study population was 2.1 (2.0), with mild liver disease diagnosed in 98.9% of patients. Mean (SD) Quan‐CCI, excluding mild liver disease, was 1.1 (2.0) (Table 1). Of the comorbidities of interest, nonalcoholic fatty liver disease (*n* = 227; 9.2%), HCC (*n* = 190; 7.7%) and cirrhosis (*n* = 153; 6.2%) were the most common. Higher proportions of patients aged ≥ 50 years had cirrhosis and HCC compared with the overall population and with patients aged < 50 years.

### 3.2 Treatment Patterns

The highest proportion of patients were indexed on a single‐agent, second‐generation NA between 2019 and 2021 (11.7%–11.9%). Of the three index treatments, entecavir (*n* = 1373; 55.5%) was the most common, followed by TAF (*n* = 895; 36.2%). The distribution of index NA therapies was numerically similar across the age groups (Table 1). Median (Q1 and Q3) time on treatment in the overall population was 40.1 (37.9, 42.4) months.

### 3.3 NA Treatment Discontinuation

One‐fifth (20.3%, *n* = 501) of patients discontinued index NA treatment, at a rate of 6.8 per 100 person‐years (95% CI 6.3–7.3), during follow‐up (Table 2, Figure S3). The proportion of patients who discontinued NA treatment increased from 10.3% at 12 months post index to 23.3% at 60 months post index. This trend was consistent across all stratifications. The mean (SD) TTD of index NA in the overall population was 20.4 (23.1) months; it was highest in the 35–49 years age group (26.0 [27.6] months).

**TABLE 2:** Patterns of NA treatment discontinuation, overall and stratified by age and index NA.

	Overall (*n* = 2473)	Age at index (in years)				Index NA		
		18–34 (*n* = 170)	35–49 (*n* = 1058)	50–64 (*n* = 1039)	65+ (*n* = 206)	Entecavir (*n* = 1373)	TAF (*n* = 895)	TDF (*n* = 205)
Discontinuation during follow‐up, *n* (%)								
No	1972 (79.7)	117 (68.8)	854 (80.7)	834 (80.3)	167 (81.1)	1026 (74.7)	788 (88.0)	158 (77.1)
Yes	501 (20.3)	53 (31.2)	204 (19.3)	205 (19.7)	39 (18.9)	347 (25.3)	107 (12.0)	47 (22.9)
Rate of discontinuation								
Rate, per 100 person‐years (95% CI)	6.77 (6.29–7.28)	9.84 (6.29–7.28)	5.93 (6.29–7.28)	7.13 (6.29–7.28)	8.41 (6.29–7.28)	7.59 (6.29–7.28)	5.02 (6.29–7.28)	5.23 (6.29–7.28)
Discontinuation at fixed time periods (cumulative), *n* (%)								
12 months, *N*	2126	151	963	856	156	1203	729	194
*n* (%)	220 (10.3)	25 (16.6)	79 (8.2)	96 (11.2)	20 (12.8)	149 (12.4)	49 (6.7)	22 (11.3)
24 months, *N*	1717	119	844	669	85	1010	532	175
*n* (%)	265 (15.4)	25 (21.0)	104 (12.3)	114 (17.0)	22 (25.9)	187 (18.5)	53 (10.0)	25 (14.3)
36 months, *N*	1347	93	693	509	52	841	351	155
*n* (%)	252 (18.7)	24 (25.8)	106 (15.3)	104 (20.4)	18 (34.6)	181 (21.5)	46 (13.1)	25 (16.1)
48 months, *N*	1049	73	568	379	29	706	210	133
*n* (%)	231 (22.0)	26 (35.6)	104 (18.3)	89 (23.5)	12 (41.4)	178 (25.2)	32 (15.2)	21 (15.8)
60 months, *N*	814	59	466	271	18	599	95	120
*n* (%)	190 (23.3)	NR	98 (21.0)	62 (22.9)	< 11	156 (26.0)	15 (15.8)	19 (15.8)
Time to discontinuation (months)								
*n*	501	53	204	205	39	347	107	47
Mean (SD)	20.4 (23.1)	20.2 (19.8)	26.0 (27.6)	16.9 (19.2)	9.2 (9.4)	21.6 (24.8)	15.5 (14.9)	22.6 (24.1)
Median (IQR)	12.2 (3.9–29.5)	11.6 (4.0–31.2)	15.2 (4.9–37.2)	9.9 (3.1–23.9)	4.4 (1.2–13.8)	12.9 (4.2–30.0)	10.8 (2.4–26.4)	13.6 (4.0–37.3)

*Note:* Primary data suppression applied to all values with fewer than 11 cases (< 11), with secondary suppression (NR) applied where required to protect primary suppression.

Abbreviations: CI, confidence interval; IQR, interquartile range; NA, nucleos(t)ide analogue; NR, not reported; SD, standard deviation; TAF, tenofovir alafenamide fumarate; TDF, tenofovir disoproxil fumarate.

The probability of treatment discontinuation at 60 months post index was numerically higher among the 18–34 years age group (39.3%; 95% CI 30.7%–49.3%) compared with the overall population and with older age groups. The probability of treatment discontinuation at 60 months post index was numerically lower in patients receiving TAF at index (18.5%; 95% CI 25.3%–30.8%) compared with the overall population and the other index NAs. The results of sensitivity analyses are presented in Tables S1 and S2.

### 3.4 NA Treatment Restart Following Discontinuation

Of the 501 patients who discontinued NA treatment, 254 (50.7%) restarted a single‐agent, second‐generation NA treatment during the follow‐up period, at a rate of 23.5 per 100 person‐years (95% CI 21.4–25.9); 14.6% (*n* = 73) of patients restarted NA treatment more than once (Table 3, Figure S4). Mean (SD) time from first discontinuation to restarting treatment was 10.9 (14.2) months. A numerically higher proportion of patients aged 35–49 years (*n* = 121; 59.3%) restarted NA treatment compared with the overall population and other age groups. A numerically lower proportion of patients receiving TAF at index restarted NA treatment following discontinuation (*n* = 42; 39.3%) compared with other index NA treatments.

**TABLE 3:** Patients who restarted NA treatment after discontinuation, overall and stratified by age and index NA.

	Overall (*n* = 501)	Age at index (in years)				Index NA		
		18–34 (*n* = 53)	35–49 (*n* = 204)	50–64 (*n* = 205)	65+ (*n* = 39)	Entecavir (*n* = 347)	TAF (*n* = 107)	TDF (*n* = 47)
Restarting NA treatment during follow‐up, *n* (%)								
No	247 (49.3)	NR	83 (40.7)	105 (51.2)	NR	157 (45.2)	65 (60.7)	25 (53.2)
Yes	254 (50.7)	NR	121 (59.3)	100 (48.8)	< 11	190 (54.8)	42 (39.3)	22 (46.8)
Restarting NA treatment over variable follow‐up								
Rate, per 100 person‐years (95% CI)	23.53 (21.42–25.86)	20.43 (15.25–27.36)	25.76 (22.63–29.32)	23.64 (20.15–27.72)	8.72 (4.36–17.43)	23.82 (21.46–26.44)	25.92 (19.8–33.93)	17.58 (12.06–25.64)
Restarting NA treatment at fixed time periods (cumulative), *n* (%)								
12 months, *N*	475	51	199	192	33	328	100	47
*n* (%)	13 (2.6)	< 11	< 11	< 11	< 11	< 11	< 11	0 (0.0)
24 months, *N*	421	44	185	166	26	297	83	41
*n* (%)	63 (12.6)	< 11	33 (16.2)	24 (11.7)	< 11	42 (12.1)	NR	< 11
36 months, *N*	348	35	162	133	18	254	57	37
*n* (%)	104 (20.8)	< 11	43 (21.1)	47 (22.9)	< 11	70 (20.2)	NR	< 11
48 months, *N*	286	31	141	102	12	221	36	29
*n* (%)	141 (28.1)	NR	57 (27.9)	66 (32.2)	< 11	97 (28.0)	33 (30.8)	11 (23.4)
60 months, *N*	228	26	123	72	< 11	188	16	24
*n* (%)	152 (30.3)	NR	65 (31.9)	67 (32.7)	< 11	104 (30.0)	34 (31.8)	14 (29.8)
Time from first discontinuation to restart (months)								
*n*	254.0	25.0	121.0	100.0	0.0	190.0	42.0	22.0
Mean (SD)	10.9 (14.2)	17.6 (20.9)	11.1 (15.1)	9.1 (10.6)	8.1 (5.5)	11.7 (15.2)	7.3 (8.0)	10.3 (13.0)
Median (IQR)	5.4 (3.4–11.7)	8.0 (3.5–21.6)	5.3 (3.2–11.7)	5.2 (3.4–9.4)	6.3 (4.1–10.9)	5.8 (3.5–12.0)	4.2 (3.2–8.5)	3.5 (3.1–16.3)
Restart NA treatment multiple times, *n* (%)								
No	428 (85.4)	NR	161 (78.9)	182 (88.8)	NR	285 (82.1)	NR	NR
Yes	73 (14.6)	< 11	43 (21.1)	23 (11.2)	NR	62 (17.9)	< 11	< 11

*Note:* Primary data suppression applied to all values with fewer than 11 cases (< 11), with secondary suppression (NR) applied where required to protect primary suppression.

Abbreviations: CI, confidence interval; IQR, interquartile range; NA, nucleos(t)ide analogue; NR, not reported; SD, standard deviation; TAF, tenofovir alafenamide fumarate; TDF, tenofovir disoproxil fumarate.

### 3.5 NA Treatment Adherence

During follow‐up, 2007 (81.2%) patients adhered (PDC ≥ 80%) to their NA treatment (Table 4). The proportion of patients who were adherent was numerically lower in the 18–34 years age group (*n* = 119, 70.0%) compared with the overall population and with other age groups. A numerically higher proportion of patients receiving TAF (*n* = 796, 88.9%) adhered to NA treatment compared with other index NAs. The proportion of patients adhering to NA treatment decreased from 87.4% to 80.7% between 12 and 60 months post index. This trend was consistent across all stratifications. The overall mean (SD) PDC over follow‐up was 0.87 (0.25) and remained generally consistent when stratified by age and index NA. Patients receiving TAF at index had the highest mean (SD) PDC (0.92 [0.20]) compared with other index NAs. The results of sensitivity analyses are presented in Table S3.

**TABLE 4:** NA treatment adherence, overall and stratified by age and index NA.

	Overall (*n* = 2473)	Age at index (in years)				Index NA		
		18–34 (*n* = 170)	35–49 (*n* = 1058)	50–64 (*n* = 1039)	65+ (*n* = 206)	Entecavir (*n* = 1373)	TAF (*n* = 895)	TDF (*n* = 205)
Adherence during follow‐up (categorical), *n* (%)								
PDC < 80%	466 (18.8)	51 (30.0)	174 (16.4)	194 (18.7)	47 (22.8)	323 (23.5)	99 (11.1)	44 (21.5)
PDC ≥ 80%	2007 (81.2)	119 (70.0)	884 (83.6)	845 (81.3)	159 (77.2)	1050 (76.5)	796 (88.9)	161 (78.5)
Adherence during follow‐up (continuous)								
Mean (SD)	0.87 (0.25)	0.80 (0.29)	0.89 (0.22)	0.87 (0.25)	0.84 (0.29)	0.84 (0.27)	0.92 (0.20)	0.86 (0.25)
Median (IQR)	0.99 (0.89–1.00)	0.96 (0.73–1.00)	0.98 (0.91–1.00)	0.99 (0.89–1.00)	0.99 (0.85–1.00)	0.98 (0.83–1.00)	1.00 (0.95–1.00)	0.98 (0.87–1.00)
Adherence at fixed time periods (cumulative), *n* (%)								
12 months, *N*	2126	151	963	856	156	1203	729	194
*n* (%)	1859 (87.4)	123 (81.5)	862 (89.5)	740 (86.4)	134 (85.9)	1013 (84.2)	675 (92.6)	171 (88.1)
24 months, *N*	1717	119	844	669	85	1010	532	175
*n* (%)	1449 (84.4)	91 (76.5)	740 (87.7)	556 (83.1)	62 (72.9)	817 (80.9)	483 (90.8)	149 (85.1)
36 months, *N*	1347	93	693	509	52	841	351	155
*n* (%)	1120 (83.1)	70 (75.3)	594 (85.7)	421 (82.7)	35 (67.3)	670 (79.7)	318 (90.6)	132 (85.2)
48 months, *N*	1049	73	568	379	29	706	210	133
*n* (%)	847 (80.7)	51 (69.9)	478 (84.2)	302 (79.7)	16 (55.2)	549 (77.8)	184 (87.6)	114 (85.7)
60 months, *N*	814	59	466	271	18	599	95	120
*n* (%)	657 (80.7)	40 (67.8)	386 (82.8)	219 (80.8)	12 (66.7)	470 (78.5)	82 (86.3)	105 (87.5)

Abbreviations: IQR, interquartile range; NA, nucleos(t)ide analogue; PDC, proportion of days covered; SD, standard deviation; TAF, tenofovir alafenamide fumarate; TDF, tenofovir disoproxil fumarate.

### 3.6 NA Treatment Switch

Overall, 16.6% (*n* = 411) of patients switched NA treatment during follow‐up (Table 5), from 4.2% (*n* = 89) at 12 months post index to 25.1% (*n* = 204) at 60 months post index. This trend was observed to be consistent for all stratifications, except in patients aged > 65 years, where the numbers for each time interval were low (only 12 patients made an NA treatment switch during follow‐up). Patients aged 18–34 years (*n* = 40; 23.5%) and patients receiving TDF as their index NA (*n* = 137; 66.8%) had the highest proportions of NA treatment switches during follow‐up. Patients receiving TAF as their index NA treatment had the lowest proportion of NA treatment switches (*n* = 35; 3.9%). Overall mean (SD) number of NA treatment switches was 2.1 (3.9), increasing from 1.2 (0.5) at 12 months post index to 1.7 (2.2) at 60 months post index. This trend was consistent for all stratifications.

**TABLE 5:** NA treatment switch, overall and stratified by age and index NA.

	Overall (*n* = 2473)	Age at index (in years)				Index NA		
		18–34 (*n* = 170)	35–49 (*n* = 1058)	50–64 (*n* = 1039)	65+ (*n* = 206)	Entecavir (*n* = 1373)	TAF (*n* = 895)	TDF (*n* = 205)
NA treatment switch during follow‐up, *n* (%)								
No	2062 (83.4)	130 (76.5)	833 (78.7)	905 (87.1)	194 (94.2)	1134 (82.6)	860 (96.1)	68 (33.2)
Yes	411 (16.6)	40 (23.5)	225 (21.3)	134 (12.9)	12 (5.8)	239 (17.4)	35 (3.9)	137 (66.8)
NA treatment switch over variable follow‐up								
Rate, per 100 person‐years (95% CI)	4.62 (4.20–5.09)	6.82 (5.00–9.30)	4.97 (4.36–5.66)	4.04 (3.41–4.79)	2.62 (1.49–4.62)	3.94 (3.47–4.48)	1.54 (1.1–2.14)	24.73 (20.92–29.24)
NA treatment switch at fixed time periods (cumulative), *n* (%)								
12 months, *N*	2126	151	963	856	156	1203	729	194
*n* (%)	89 (4.2)	NR	40 (4.2)	34 (4.0)	< 11	44 (3.7)	13 (1.8)	32 (16.5)
24 months, *N*	1717	119	844	669	85	1010	532	175
*n* (%)	164 (9.6)	NR	84 (10.0)	57 (8.5)	< 11	80 (7.9)	22 (4.1)	62 (35.4)
36 months, *N*	1347	93	693	509	52	841	351	155
*n* (%)	203 (15.1)	NR	114 (16.5)	65 (12.8)	< 11	101 (12.0)	20 (5.7)	82 (52.9)
48 months, *N*	1049	73	568	379	29	706	210	133
*n* (%)	208 (19.8)	NR	116 (20.4)	65 (17.2)	< 11	104 (14.7)	17 (8.1)	87 (65.4)
60 months, *N*	814	59	466	271	18	599	95	120
*n* (%)	204 (25.1)	NR	123 (26.4)	57 (21.0)	< 11	104 (17.4)	< 11	NR
Number of NA treatment switches during follow‐up (continuous)								
Mean (SD)	2.1 (3.9)	2.7 (5.8)	2.3 (4.1)	1.7 (2.7)	1.3 (0.9)	2.6 (4.7)	2.4 (2.3)	1.3 (2.0)
Median (IQR)	1.0 (1.0–2.0)	1.0 (1.0–2.0)	1.0 (1.0–2.0)	1.0 (1.0–1.0)	1.0 (1.0–1.0)	1.0 (1.0–2.0)	2.0 (1.0–2.0)	1.0 (1.0–1.0)

*Note:* Primary data suppression applied to all values with fewer than 11 cases (< 11), with secondary suppression (NR) applied where required to protect primary suppression.

Abbreviations: CI, confidence interval; IQR, interquartile range; NA, nucleos(t)ide analogue; NR, not reported; SD, standard deviation; TAF, tenofovir alafenamide fumarate; TDF, tenofovir disoproxil fumarate.

### 3.7 Factors Associated With Treatment Discontinuation and Adherence

**FIGURE 1:**
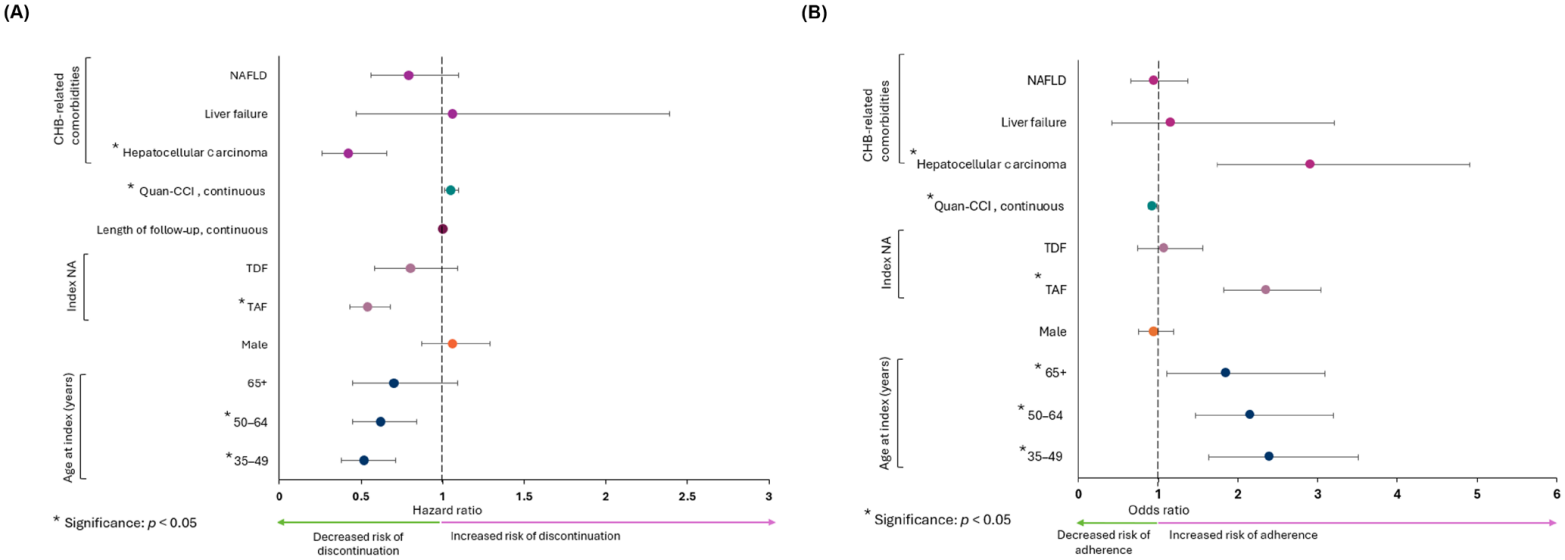
Factors associated with (A) treatment discontinuation and (B) adherence. CCI, Charlson comorbidity index; CHB, chronic hepatitis B; NA, nucleos(t)ide analogue; NAFLD, nonalcoholic fatty liver disease; TAF, tenofovir alafenamide fumarate; TDF, tenofovir disoproxil fumarate.

Factors significantly associated with a decreased risk of discontinuing NA treatment included patients receiving TAF as their index NA treatment (vs. entecavir) (HR 0.58; 95% CI 0.46–0.72), patients with HCC diagnosis at baseline (HR 0.42; 95% CI 0.26–0.66), and patients aged 35–64 years (vs. 18–34 years) (HR [35–49 years] 0.52; 95% CI 0.38–0.70; HR [50–64 years] 0.63; 95% CI 0.46–0.86) (Figure 1A). A higher Quan‐CCI score was significantly associated with an increased risk of discontinuing NA treatment (HR 1.06; 95% CI 1.01–1.10).

Factors significantly associated with increased odds of adherence included age (≥ 35 years vs. 18–34 years) (OR [35–49 years] 2.36; 95% CI 1.61–3.46; OR [50–64 years] 2.08; 95% CI 1.41–3.08; OR [> 65 years] 1.74; 95% CI 1.04–2.91), TAF at index (vs. entecavir) (OR 2.42; 95% CI 1.85–3.16) and having an HCC diagnosis at baseline (OR 2.81; 95% CI 1.68–4.71) (Figure 1B). A higher Quan‐CCI score was significantly associated with decreased probability of adherence (OR 0.92; 95% CI 0.87–0.96).

### 3.8 Possible Reasons for Index NA Treatment Discontinuation

Among the patients who discontinued treatment (*n* = 501), 69.3% (*n* = 347) did so for possible physician‐guided decisions; this was observed in a lower proportion among patients aged 18–34 years (*n* = 28; 52.8%) than those aged ≥ 35 years. Distribution between possible physician‐ and non‐physician‐guided reasons for discontinuation was similar when stratified by index NA.

## 4 Discussion

Second‐generation NAs are a recommended treatment option for patients with CHB, but persistence with and adherence to treatment are important to achieve sustained viral suppression. This study offers valuable insights into the treatment patterns and factors influencing the risk of discontinuation and adherence to second‐generation NA therapy in Japan. One‐fifth of patients discontinued NA treatment, despite Japan's implementation of comprehensive measures for hepatitis control and a nationwide hepatitis awareness campaign (the ‘Shitte kan‐en’ project) [4, 25, 26]; of these, half restarted NA treatment during the follow‐up period. Approximately 80% of the overall population were adherent to their index NA treatment. Factors significantly associated with persistence and adherence included TAF initiation at index and the presence of baseline HCC.

Treatment discontinuation increased over time, consistent with previous research [21, 27], possibly owing to attainment of the short‐term goals of antiviral therapy: HBeAg seroconversion in patients with HBeAg‐positive CHB, persistent normalisation of alanine transaminase < 30 U/L and suppression of HBV DNA replication [9, 28]. Other possible reasons for discontinuation could be side effects associated with the treatment [11, 29] or a switch to other antiviral therapies such as pegylated interferon [9, 29]. This is corroborated by the observation that a physician‐guided decision was identified as a possible reason for treatment discontinuation in the majority of patients across all stratifications except those aged 18–34 years.

A numerically higher proportion of patients aged 18–34 years discontinued treatment. This could be attributed to physicians being hesitant to maintain younger patients on long‐term treatment [7]. However, indefinite NA therapy for CHB is preferred by most patients and physicians to prevent viral relapse and minimise the need for intensive treatment monitoring [30]. Patients receiving TAF at index had the lowest proportion of treatment discontinuation and the highest treatment adherence; this might be because TAF is better tolerated, is easier to administer (can be taken with food vs. the empty stomach required for entecavir) [31], and is associated with a lower risk of renal‐function decline compared with entecavir [16, 32, 33].

Half of the patients who discontinued treatment restarted NA therapy during their available follow‐up, perhaps because of reactivation of disease [9]. Patients receiving TDF at index had a higher proportion of NA treatment switches during follow‐up compared with the other stratifications. This might be due to the clinical guidance recommendations for switching from TDF if it is not well tolerated [9].

NA treatment adherence observed in this study aligned with that observed in claims‐based studies from other countries [21, 34, 35]. Adherence was lowest in the 18–34 years age group, consistent with the observation of poor adherence to medication in many clinical conditions among adolescents and young adults, often attributed to their low recognition of treatment benefits [21]. Patients who received TAF at index were observed to be more adherent to the treatment, possibly due to its easier dosing [31, 33] and better safety profile with regard to decreased bone mineral density loss and renal toxicity compared with the other NAs, which is especially important in an ageing population that has other comorbidities [36]. However, despite studies suggesting that TAF has a better safety profile than TDF, more real‐world studies are needed to establish its long‐term safety [16, 37]. Consistent with the literature, overall adherence decreased over time across all stratifications, possibly because the long‐term nature of the therapy, which usually starts at a young age, results in disengagement from clinical care [37, 38].

Entecavir was the most common index NA treatment in our study. This is consistent with it being the first second‐generation NA to receive approval in Japan [9]. A decrease in NA uptake was observed briefly in 2013, possibly due to the 2013 updates to the Japan Society of Hepatology guidelines recommending interferon therapy as the first‐line treatment for CHB [7], which may have temporarily altered prescribing patterns while only one second‐generation NA was available in Japan (i.e., entecavir).

Results from a similar study conducted in the US have recently been reported [35]. Although the proportion of adherent patients (87%) was also high, there was a marked difference in the proportion who discontinued NA treatment (41% vs. 20% in this study). Among the differences between the two studies were the lower proportion of men in the US study (58% vs. 66%) and the most common NA treatment at index being TAF (48%) in the US study (vs. entecavir [55%] in the Japan study). Of the patients who discontinued their index NA treatment, a higher proportion in the Japan study did so under possible physician guidance (69.3% vs. 12.6% in the US study). However, direct comparisons of discontinuation between the two countries are limited by differences in medical systems, such as variations in the follow‐up lab tests used to guide treatment decisions. The higher rate of physician‐guided treatment discontinuation in Japan may indicate more active monitoring of NA‐treated patients, which might also contribute to the smaller proportion of patients discontinuing treatment compared with the US study. The higher rates of persistence and physician‐guided discontinuation may also be due to reduced financial barriers to NA therapy under Japan's universal healthcare system [4], education and strong national awareness about the risks of CHB [26], as well as a healthcare culture in Japan in which patients tend to follow medical advice [39].

In this study, the peri‐ and post‐pandemic periods had lower proportions of treatment discontinuation and higher proportions of adherence than the pre‐pandemic period. Healthcare restrictions during the peri‐pandemic period permitted patients to utilise repeat prescription services; this behaviour possibly continued in the post‐pandemic period [40], thereby improving persistence and adherence to NA therapy.

Limitations of this study include that medical records collected in the JMDC Inc. database are restricted to patients in employment, potentially meaning they are not representative of the real‐world CHB patient population, particularly elderly patients. Although the second‐generation NA users represented in this study reflect the current clinical landscape, the study findings cannot be generalised to lamivudine and adefovir users. In addition, the study used information from filled prescriptions, which may not represent the actual use of medications, thereby overestimating adherence. NA use for prevention of disease reactivation may also have been included in the analysis. Further, identifying reasons for NA discontinuation in this study were based on the presence of claims for laboratory test orders (HBV antigen, HBV DNA and liver function tests) and did not capture all possible reasons leading to treatment discontinuation, such as patients completing immunosuppressive therapies or chemotherapies. Finally, time‐to‐event analyses assume censoring to be noninformative. However, informative censoring in this study, such as coinfection, when the patient was no longer treated with single‐agent NA, could be directly related to the outcomes of interest.

In conclusion, the results of this study highlight treatment discontinuation as a significant challenge among patients undergoing long‐term NA therapy for CHB, particularly within the younger population. The study shows that there is a critical need for finite therapies, with shorter treatment durations and less stringent monitoring, capable of achieving a functional cure for CHB. Such novel treatments could improve persistence to therapy and enhance the clinical outcomes for patients with CHB.

## Author Contributions

**Shinya Kawamatsu:** conception or design, data analysis and interpretation. **Kiran K. Rai:** conception or design, data analysis and interpretation. **Vera Gielen:** conception or design. **Shayon Salehi:** conception or design, acquisition of data (e.g., study investigator), data analysis and interpretation. **Amisha Patel:** conception or design, data analysis and interpretation. **Olivia Massey:** conception or design, data analysis and interpretation. **Seth W. Anderson:** conception or design. **Yutaka Handa:** conception or design, data analysis and interpretation. **Ethan Yichen Lee:** conception or design, data analysis and interpretation. **Poppy Payne:** conception or design, data analysis and interpretation. **Isabel Jimenez:** conception or design, data analysis and interpretation. **Kejsi Begaj:** data analysis and interpretation. **Jun Inoue:** data analysis and interpretation. **Afisi S. Ismaila:** data analysis and interpretation. All named authors meet the International Committee of Medical Journal Editors (ICMJE) criteria for authorship for this article, take responsibility for the integrity of the work as a whole, and have given their approval for this version to be published.

## Ethics Statement

This study complied with all applicable laws regarding subject privacy. No direct subject contact or primary collection of individual human subject data occurred. As the Japanese Medical Data Center Claims Database data are anonymized in accordance with Clause 2:9 of the Law for the Protection of Personal Information, and the individuals cannot be identified, its use is exempt from broad institutional review board approval.

## Consent

The authors have nothing to report.

## Conflicts of Interest

K.K.R., A.P., P.P., I.J. and O.M. are employees of Adelphi Real World; Adelphi Real World received funding from GSK to conduct the study. A.S.I., S.K., Y.H., E.Y.L. and S.S. are employees of or hold financial equities in GSK. A.S.I. is also an unpaid part‐time member of the McMaster University faculty. S.W.A. and V.G. were employees of or held financial equities in GSK at the time of the study. J.I. declares no conflicts of interest in this study. K.B. is a post‐doctoral university worker employed by GSK through the Center of Health Outcomes, Policy and Economics at Rutgers University.

## Supporting information


**Appendix S1:** jvh70062‐sup‐0001‐AppendixS1.docx.

## Data Availability

The data that support the findings of this study are available from the corresponding author upon reasonable request.
