# Supplementary material for: Treatment Discontinuation and Adherence in Patients With Chronic Hepatitis B Infection Newly Initiating Nucleos(t)ide Analogues in Japan: A Retrospective Cohort Study
Source: J Viral Hepat. 2025 Aug 14;32(9):e70062. doi: 10.1111/jvh.70062 (PMC12351530; doi:10.1111/jvh.70062)
Supplement: Supplementary file 1 — Appendix S1: jvh70062‐sup‐0001‐AppendixS1.docx. [file JVH-32-0-s001.docx]

# Supplemental materials

**FIGURE S1** Study design.

**FIGURE S2** Patient attrition flow chart.

**FIGURE S3** Kaplan–Meier estimates (survival function), presented for time to discontinuation.

FIGURE S4 Kaplan–Meier estimates (survival function), presented for time to restarting single-agent, second-generation NA following first discontinuation.

**TABLE S1** Sensitivity analysis (≥60-day permissible gap) to assess discontinuation of newly initiated NA treatment, stratified by age, index NA prescriber specialty, and index NA.

**TABLE S2** Sensitivity analysis in the pre-, peri-, and post-pandemic periods to assess discontinuation of newly initiated NA treatment, stratified by age, index NA prescriber specialty, and index NA.

**TABLE S3** Sensitivity analysis in the pre-, peri-, and post-pandemic periods to assess adherence to newly initiated NA treatment, stratified by age, index NA prescriber specialty, and index NA.

**FIGURE S1** Study design.

CHB, chronic hepatitis B; HCV, hepatitis C virus; HDV, hepatitis D virus; HIV, human immunodeficiency virus; NA, nucleos(t)ide analog.


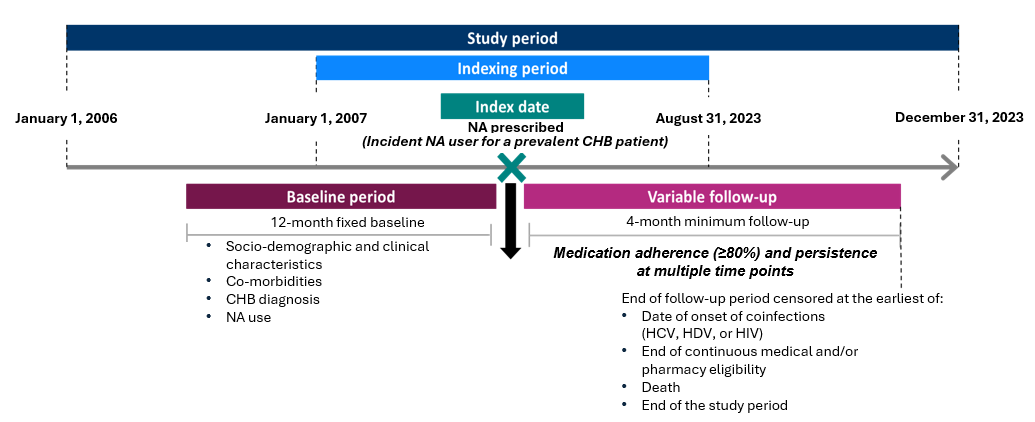


**FIGURE S2** Patient attrition flow chart.

*Laboratory-confirmed CHB diagnosis; ⴕPatients censored from outcomes assessment when meeting the earliest of the following criteria: date of onset of coinfections (HCV, HDV, or HIV), end of continuous medical and pharmacy eligibility, death, or end of study period (i.e., December 31, 2023, or latest data available).

CHB, chronic hepatitis B; HCV, hepatitis C virus; HDV, hepatitis D virus; HIV, human immunodeficiency virus; NA, nucleos(t)ide analog; TAF, tenofovir alafenamide fumarate; TDF, tenofovir disoproxil fumarate.

All patients receiving first- and second-generation NAs, and any combinations associated with NAs between January 1, 2007, and August 31, 2023 (i.e., data cut)
***n* = 18,665**

At least one pharmacy claim for a single-agent, second-generation NA between January 1, 2007, and August 31, 2023
***n* = 13,375**

Aged ≥18 years on or prior to index date
***n* = 13,368**

^*^At least one diagnosis claim for CHB infection (inpatient or outpatient) during the 12-month baseline period
***n* = 8120**

At least 4 months (120 days) of continuous enrollment in claims data following and including index date
***n* = 7485**

At least 12 months of continuous enrollment in claims data prior to and excluding index date
***n*  = 2875**

No diagnosis claim for coinfection (HCV, HDV, HIV) in any setting during the 12-month baseline period
***n* = 2785**

No pharmacy claim for a first- or second-generation NA (lamivudine, adefovir dipivoxil, entecavir, TDF, TAF) during the 12-month baseline period
***n* = 2487**

^ⴕ^No onset of coinfection, end of continuous medical and pharmacy eligibility, or death within the 4-month follow-up period
***n* = 2480**

No pharmacy claims for two or more different second-generation NAs on the index date
***n* =** **2473**

**n=30,165**

*5290 people excluded*

*7 people excluded*

*5248 people excluded*

*635 people excluded*

*4610 people excluded*

*90 people excluded*

*298 people excluded*

*7 people excluded*

*7 people excluded*

**FIGURE S3** Kaplan–Meier estimates (survival function), presented for time to discontinuation. a) Overall cohort, b) stratified by age at index (years), c) stratified by index NA.

NA, nucleos(t)ide analog; TAF, tenofovir alafenamide fumarate; TDF, tenofovir disoproxil fumarate.

**
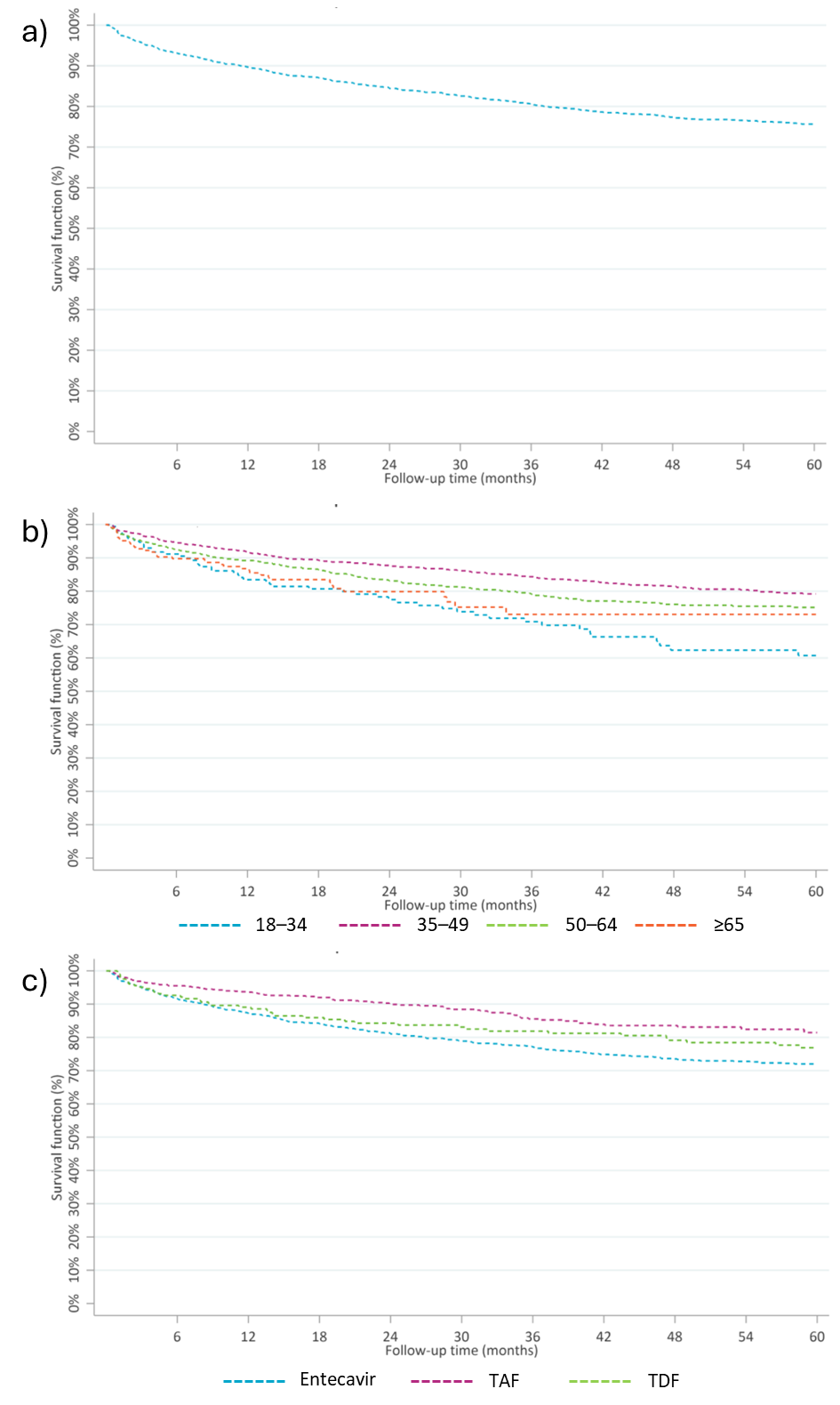
**

FIGURE S4 Kaplan–Meier estimates (survival function), presented for time to restarting single-agent, second-generation NA following first discontinuation. a) Overall cohort, b) stratified by age at index (years), c) stratified by index NA.

NA, nucleos(t)ide analog; TAF, tenofovir alafenamide fumarate; TDF, tenofovir disoproxil fumarate.


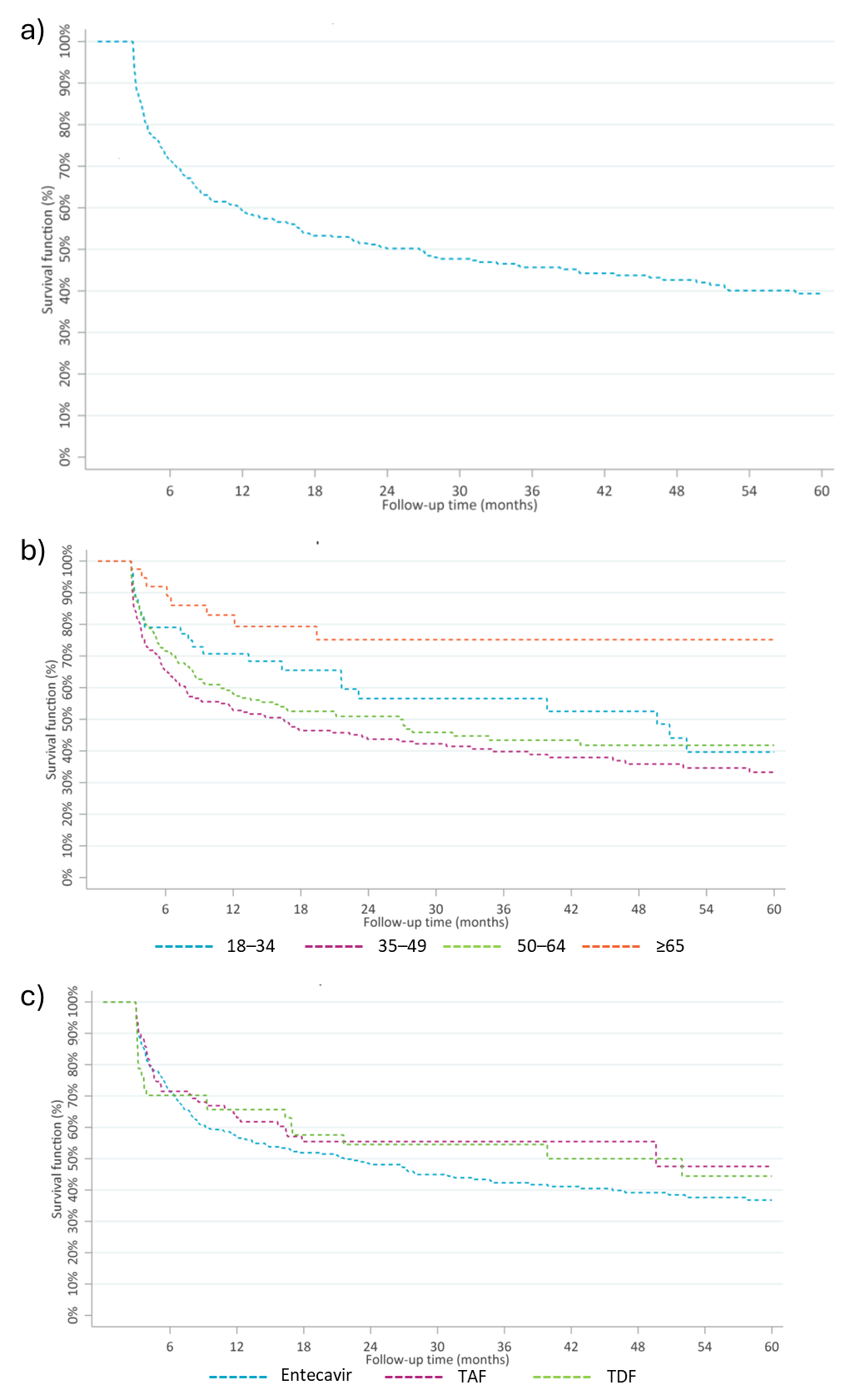


**TABLE S1** Sensitivity analysis (≥60-day permissible gap) to assess discontinuation of newly initiated NA treatment, stratified by age, index NA prescriber specialty, and index NA.

|  | **Age at index** | | | | | **Indexed NA** | | |
| --- | --- | --- | --- | --- | --- | --- | --- | --- |
|  | **Overall** | **18–34 years** | **35–49 years** | **50–64 years** | **≥65 years** | **Entecavir** | **TAF** | **TDF** |
|  | **(*n* = 2473)** | **(*n* = 170)** | **(*n* = 1058)** | **(*n* = 1039)** | **(*n* = 206)** | **(*n* = 1373)** | **(*n* = 895)** | **(*n* = 205)** |
| **Discontinuation in follow-up, *n***  No  Yes | 2473  1813 (73.3)  660 (26.7) | 170  105 (61.8)  65 (38.2) | 1058  772 (73.0)  286 (27.0) | 1039  782 (75.3)  257 (24.7) | 206  154 (74.8)  52 (25.2) | 1373  931 (67.8)  442 (32.2) | 895  736 (82.2)  159 (17.8) | 205  146 (71.2)  59 (28.8) |
| **Discontinuation (variable follow-up) in months** | | | | | | | | |
| Total exposures  Total exposure time  Rate (per 100 person-years)  95% CI | 1102  3,808,334  10.53  9.93–^†^11.17 | 131  280,997  16.97  14.3–20.14 | 511  1,981,169  9.39  8.60–10.24 | 392  1,373,004  10.39  9.41–11.47 | 68  173,164  14.29  11.27–18.13 | 821  2,554,917  11.7  10.92–12.52 | 200  863,605  8.43  7.34–9.68 | 81  389,812  7.56  6.08–9.40 |
| **Discontinuation at fixed time periods (cumulative), *n* (%)** | | | | | | | | |
| *n*  12 months | 2126  266 (12.5) | 151  30 (19.9) | 963  101 (10.5) | 856  112 (13.1) | 156  23 (14.7) | 1203  182 (15.1) | 729  59 (8.1) | 194  25 (12.9) |
| *n*  24 months | 1717  339 (19.7) | 119  31 (26.1) | 844  140 (16.6) | 669  138 (20.6) | 85  30 (35.3) | 1010  237 (23.5) | 532  71 (13.3) | 175  31 (17.7) |
| *n*  36 months | 1347  324 (24.1) | 93  32 (34.4) | 693  142 (20.5) | 509  127 (25.0) | 52  23 (44.2) | 841  235 (27.9) | 351  57 (16.2) | 155  32 (20.6) |
| *n*  48 months | 1049  294 (28.0) | 73  30 (41.1) | 568  140 (24.6) | 379  108 (28.5) | 29  16 (55.2) | 706  222 (31.4) | 210  45 (21.4) | 133  27 (20.3) |
| *n*  60 months | 814  242 (29.7) | 59  NR | 466  130 (27.9) | 271  77 (28.4) | 18  <11 | 599  196 (32.7) | 95  20 (21.1) | 120  26 (21.7) |
| **TTD, days** | | | | | | | | |
| *n*  Mean (SD)  Median  IQR (min, max) | 2473  39.5 (35.9)  28.5  12.2–55.9  (0.3, 199.5) | 170  36.3 (35.7)  24.6  10.7–48.0  (0.6, 162.9) | 1058  48.7 (39.8)  37.1  17.9–70.2  (0.3, 199.5) | 1039  34.5 (31.4)  25.0  10.3–49.7  (0.3, 179.0) | 206  20.6 (19.1)  15.2  8.7–25.2  (0.3, 113.8) | 1373  45.2 (42.2)  29.6  11.6–68.8  (0.3, 199.5) | 895  28.1 (18.9)  25.1  11.9–41.9  (0.3, 78.7) | 205  51.2 (33.6)  52.5  17.7–81.3  (1.0, 111.2) |
| **Probability of TTD (Kaplan–Meier estimate)**  **TTD, months** | | | | | | | | |
| Censored  Events  25% survival time  95% CI  Median survival time  95% CI | 3  2470  42.61  38.27–48.62  132.39  116.85–139.91 | 0  170  23  13.11–35.38  77.1  46.81, –^†^ | 2  1056  56.34  48.19–65.31  139.45  125.59–142.41 | 1  1038  40.37  33.97–47.86  132.39  116.69, –^†^ | 0  206  28.58  13.83–40.34  106.64  49.24, –^†^ | 1  1372  36.2  29.70–41.85  132.39  116.43–139.82 | 1  894  48.92  40.93–56.14  0  70.17, –^†^ | 1  204  58.51  32.13–83.15  0  95.93, –^†^ |
| **12 months post index**  No. fail  No. at risk  Failure function  95% CI | 120  1901  0.111  0.099–0.124 | 12  126  0.177  0.126–0.245 | 46  880  0.087  0.126–0.245 | 52  760  0.116  0.126–0.245 | 10  135  0.154  0.126–0.245 | 76  1051  0.136  0.119–0.155 | 34  679  0.072  0.119–0.155 | 10  171  0.109  0.119–0.155 |
| **24 months post index**  No. fail  No. at risk  Failure function  95% CI | 73  1446  0.172  0.157–0.189 | 7  93  0.262  0.199–0.339 | 34  737  0.137  0.199–0.339 | 26  553  0.184  0.199–0.339 | 6  63  0.231  0.199–0.339 | 42  820  0.203  0.182–0.226 | 26  477  0.126  0.182–0.226 | 5  149  0.163  0.182–0.226 |
| **36 months post index**  No. fail  No. at risk  Failure function  95% CI | 62  1090  0.219  0.201–0.238 | 9  69  0.324  0.254–0.409 | 26  584  0.181  0.254–0.409 | 26  403  0.228  0.254–0.409 | 1  34  0.328  0.254–0.409 | 40  657  0.247  0.223–0.273 | 16  304  0.184  0.223–0.273 | 6  129  0.192  0.223–0.273 |
| **48 months post index**  No. fail  No. at risk  Failure function  95% CI | 33  816  0.268  0.248–0.290 | 2  46  0.426  0.342–0.522 | 21  463  0.22  0.342–0.522 | 8  290  0.284  0.342–0.522 | 2  17  0.352  0.342–0.522 | 17  527  0.296  0.270–0.325 | 13  178  0.237  0.270–0.325 | 3  111  0.232  0.270–0.325 |
| **60 months post index**  No. fail  No. at risk  Failure function  95% CI | 104  623  0.301  0.279–0.324 | 6  36  0.454  0.366–0.552 | 67  367  0.259  0.366–0.552 | 28  209  0.306  0.366–0.552 | 3  11  0.444  0.366–0.552 | 85  443  0.32  0.292–0.350 | 7  80  0.314  0.292–0.350 | 12  100  0.253  0.292–0.350 |

^†^Lower and/or upper bounds are outside of the observable range (i.e., follow-up).

Primary data suppression applied to all values with fewer than 11 cases (<11), with secondary suppression (NR) applied where required to protect primary suppression.

Abbreviations: CI, confidence interval; IQR, interquartile range; NA, nucleos(t)ide analog; NR, not recorded; SD, standard deviation; TAF, tenofovir alafenamide; TDF, tenofovir disoproxil fumarate; TTD, time to discontinuation.

## Sensitivity analysis of NA treatment discontinuation

When a treatment gap of ≥60 days was considered, the proportion of patients who discontinued their index NA treatment was higher than that observed in the main analysis (26.7% vs. 20.3%). The same trend was observed across stratifications. Mean (SD) TTD of index NA was also higher compared with the main analysis (39.5 [35.9] vs. 20.4 [23.1] months).

**TABLE S2.** Sensitivity analysis in the pre-, peri-, and post-pandemic periods to assess discontinuation of newly initiated NA treatment, stratified by age, index NA prescriber specialty, and index NA. Overall, a numerically higher proportion of patients discontinued index NA treatment in the pre-pandemic period (*n* = 264; 16.9%) compared with the peri- (*n* = 21; 4.8%) and post-pandemic (*n* = 36; 7.5%) periods.

| **SENSITIVITY ANALYSIS: Pre-pandemic period (for patients indexed prior to and excluding April 7, 2020)** | | | | | | | | |
| --- | --- | --- | --- | --- | --- | --- | --- | --- |
|  |  | **Age at index** | | | | **Indexed NA** | | |
|  | **Overall**  **(*n* = 1558)** | **18–34 years**  **(*n* = 109)** | **35–49 years**  **(*n* = 712)** | **50–64 years**  **(*n* = 630)** | **≥65 years**  **(*n* = 107)** | **Entecavir**  **(*n* = 1035)** | **TAF**  **(*n* = 333)** | **TDF**  **(*n* = 190)** |
| **Discontinuation in follow-up, *n* (%)**  No  Yes | 1294 (83.1)  264 (16.9) | 78 (71.6)  31 (28.4) | 603 (84.7)  109 (15.3) | 526 (83.5)  104 (16.5) | 87 (81.3)  20 (18.7) | 814 (78.6)  221 (21.4) | 318 (95.5)  15 (4.5) | 162 (85.3)  28 (14.7) |
| **Discontinuation (variable follow-up), months** | | | | | | | | |
| Total exposures  Total exposure time  Rate (per 100 person-years)  95% CI | 348  3,196,886  3.96  3.57–4.40 | 41  241,069  6.19  4.56–8.41 | 157  1,726,852  3.31  2.83–3.87 | 130  1,112,558  4.25  3.58–5.05 | 20  116,407  6.25  4.03–9.69 | 303  2,325,350  4.74  4.24–5.31 | 15  493,484  1.11  0.67–1.84 | 30  378,052  2.89  2.02–4.13 |
| **Discontinuation at fixed time periods (cumulative), *n* (%)** | | | | | | | | |
| *n*  12 months | 1459  141 (9.7) | 102  15 (14.7) | 695  55 (7.9) | 575  59 (10.3) | 87  12 (13.8) | 956  112 (11.7) | 322  14 (4.3) | 181  15 (8.3) |
| *n*  24 months | 1324  175 (13.2) | 95  18 (18.9) | 663  73 (11.0) | 508  70 (13.8) | 58  14 (24.1) | 862  143 (16.6) | 294  13 (4.4) | 168  19 (11.3) |
| *n*  36 months | 1198  174 (14.5) | 85  19 (22.4) | 625  74 (11.8) | 445  70 (15.7) | 43  11 (25.6) | 784  145 (18.5) | 264  <11 | 150  NR |
| *n*  48 months | 1049  175 (16.7) | 73  NR | 568  78 (13.7) | 379  67 (17.7) | 29  <11 | 706  149 (21.1) | 210  <11 | 133  NR |
| *n*  60 months | 814  156 (19.2) | 59  NR | 466  78 (16.7) | 271  52 (19.2) | 18  <11 | 599  134 (22.4) | 95  <11 | 120  NR |
| **TTD, days** | | | | | | | | |
| *n*  Mean (SD)  Median  IQR (min, max) | 264  17.2 (19.5)  10.2  3.3–24.0  (0.3, 111.1) | 31  20.0 (20.3)  11.6  3.3–32.4  (1.0, 77.1) | 109  21.0 (23.5)  11.9  4.5–31.3  (0.3, 111.1) | 104  14.0 (14.7)  8.8  2.7–21.2  (0.3, 83.6) | 20  8.8 (8.9)  4.3  1.7–13.5  (1.0, 29.5) | 221  18.5 (20.3)  11.5  4.2–26.2  (0.3, 111.1) | 15  3.4 (4.6)  1.7  1.0–3.9  (0.4, 17.7) | 28  14.3 (14.7)  10.0  2.8–20.5  (1.0, 57.3) |
| **Probability of TTD (Kaplan–Meier estimate)** | | | | | | | | |
| **TTD, months** | | | | | | | | |
| Censored  Events  25% survival time  95% CI  Median survival time  95% CI | 2  1556  66.89  46.19–99.51  0  0.00–0.00 | 0  109  31.18  13.57–46.81  0  46.81, –^†^ | 1  711  78.61  66.82, –^†^  0  0.00–0.00 | 1  629  83.57  37.52, –^†^  0  0.00–0.00 | 0  107  28.81  12.19, –^†^  0  0.00–0.00 | 0  1035  46.19  36.86–72.40  0  0.00–0.00 | 1  332  0  0.00–0.00  0  0.00–0.00 | 1  189  0  43.46, –^†^  0  0.00–0.00 |
| **12 months post index**  No. fail  No. at risk  Failure function  95% CI | 54  1042  0.103  0.088–0.120 | 4  71  0.158  0.100–0.246 | 21  509  0.087  0.100–0.246 | 24  411  0.106  0.100–0.246 | 5  51  0.139  0.100–0.246 | 46  741  0.122  0.103–0.144 | 1  148  0.044  0.103–0.144 | 7  153  0.081  0.103–0.144 |
| **24 months post index**  No. fail  No. at risk  Failure function  95% CI | 24  706  0.156  0.137–0.177 | 5  51  0.212  0.141–0.312 | 7  375  0.128  0.141–0.312 | 10  256  0.168  0.141–0.312 | 2  24  0.231  0.141–0.312 | 21  555  0.181  0.158–0.209 | 0  23  0.054  0.158–0.209 | 3  128  0.125  0.158–0.209 |
| **36 months post index**  No. fail  No. at risk  Failure function  95% CI | 21  501  0.189  0.166–0.213 | 4  34  0.306  0.212–0.427 | 10  283  0.146  0.212–0.427 | 7  170  0.205  0.212–0.427 | 0  14  0.301  0.212–0.427 | 19  419  0.216  0.189–0.246 | 0  0  0  0.189–0.246 | 2  82  0.15  0.189–0.246 |
| **48 months post index**  No. fail  No. at risk  Failure function  95% CI | 5  345  0.228  0.201–0.257 | 0  24  0.398  0.285–0.535 | 5  205  0.18  0.285–0.535 | 0  107  0.243  0.285–0.535 | 0  9  0.301  0.285–0.535 | 4  310  0.255  0.225–0.289 | 0  0  0  0.225–0.289 | 1  35  0.178  0.225–0.289 |
| **60 months post index**  No. fail  No. at risk  Failure function  95% CI | 13  260  0.24  0.212–0.270 | 2  19  0.398  0.285–0.535 | 10  154  0.201  0.285–0.535 | 1  81  0.243  0.285–0.535 | 0  6  0.301  0.285–0.535 | 13  252  0.265  0.234–0.300 | 0  0  0  0.234–0.300 | 0  8  0.224  0.234–0.300 |
|  | | | | | | | | |
| **SENSITIVITY ANALYSIS: Peri-pandemic period (for patients indexed from April 7, 2020, to September 30, 2021)** | | | | | | | | |
|  |  | **Age at index** | | | | **Indexed NA** | | |
|  | **Overall**  **(*n* = 434)** | **18–34 years**  **(*n* = 31)** | **35–49 years**  **(*n* = 180)** | **50–64 years**  **(*n* = 184)** | **≥65 years**  **(*n* = 39)** | **Entecavir**  **(*n* = 165)** | **TAF**  **(*n* = 260)** | **TDF**  **(*n* = 9)** |
| **Discontinuation in follow-up, *n* (%)**  No  Yes | 434  413 (95.2)  21 (4.8) | 31  NR  <11 | 180  NR  <11 | 184  NR  <11 | 39  NR  <11 | 165  154 (93.3)  11 (6.7) | 260  NR  <11 | 9  <11  <11 |
| **Discontinuation (variable follow-up), months** | | | | | | | | |
| Total exposures  Total exposure time  Rate (per 100 person-years)  95% CI | 21  402,150  1.9  1.24–2.92 | 3  25,661  4.26  1.37–13.19 | 8  177,399  1.64  0.82–3.28 | 7  168,544  1.51  0.72–3.17 | 3  30,546  3.57  1.15–11.08 | 11  154,177  2.6  1.44–4.69 | 7  238,572  1.07  0.51–2.24 | 3  9401  11.62  3.75–36.02 |
| **Discontinuation at fixed time periods (cumulative), *n* (%)** | | | | | | | | |
| *n*  12 months | 392  18 (4.6) | 28  <11 | 171  <11 | 162  <11 | 31  <11 | 149  <11` | 234  <11 | 9  <11 |
| *n*  24 months | 349  16 (4.6) | 19  <11 | 159  <11 | 148  <11 | 23  <11 | 134  <11 | 208  <11 | 7  <11 |
| *n*  36 months | 149  13 (8.7) | 8  <11 | 68  <11 | 64  <11 | 9  <11 | 57  <11 | 87  <11 | 5  <11 |
| **TTD, days** | | | | | | | | |
| *n*  Mean (SD)  Median  IQR (min, max) | 21.0  3.4 (3.1)  2.4  0.9–4.7  (0.3, 9.5) | 3.0  6.0 (3.1)  7.8  2.4–7.8  (2.4, 7.8) | 8.0  2.5 (1.7)  2.5  0.9–4.2  (0.3, 4.7) | 7.0  3.4 (3.9)  0.9  0.5–8.0  (0.3, 9.5) | 3.0  3.4 (4.3)  1.0  0.9–8.4  (0.9, 8.4) | 11.0  3.7 (3.5)  3.0  0.7–8.0  (0.3, 9.5) | 7.0  2.7 (2.6)  2.0  0.6–4.2  (0.5, 7.8) | 3.0  4.2 (3.4)  4.0  1.0–7.8  (1.0, 7.8) |
| **Probability of TTD (Kaplan–Meier estimate)** | | | | | | | | |
| **TTD, months** | | | | | | | | |
| Censored  Events  25% survival time  95% CI  Median survival time  95% CI | 1  433  0  0.00–0.00  0  0.00–0.00 | 0  31  0  2.40, –^†^  0  0.00–0.00 | 1  179  0  0.00–0.00  0  0.00–0.00 | 0  184  0  0.00–0.00  0  0.00–0.00 | 0  39  0  8.38, –^†^  0  0.00–0.00 | 1  164  0  0.00–0.00  0  0.00–0.00 | 0  260  0  0.00–0.00  0  0.00–0.00 | 0  9  7.75  0.95, –^†^  0  0.00–0.00 |
| **12 months post index**  No. fail  No. at risk  Failure function  95% CI | 0  102  0.067  0.043–0.103 | 0  8  0.153  0.051–0.408 | 0  41  0.053  0.051–0.408 | 0  44  0.056  0.051–0.408 | 0  9  0.108  0.051–0.408 | 0  33  0.097  0.053–0.175 | 0  67  0.034  0.053–0.175 | 0  2  0.429  0.053–0.175 |
|  | | | | | | | | |
| **SENSITIVITY ANALYSIS: Post-pandemic period (for patients indexed from October 1, 2021, to August 31, 2023)** | | | | | | | | |
|  | **Age at index** | | | | | **Indexed NA** | | |
|  | **Overall**  **(*n* = 481)** | **18–34 years**  **(*n* = 30)** | **35–49 years**  **(*n* = 166)** | **50–64 years**  **(*n* = 225)** | **≥65 years**  **(*n* = 60)** | **Entecavir**  **(*n* = 173)** | **TAF**  **(*n* = 302)** | **TDF**  **(*n* = 6)** |
| **Discontinuation in follow-up, *n* (%)**  No  Yes | 481  445 (92.5)  36 (7.5) | 30  NR  <11 | 166  NR  <11 | 225  207 (92.0)  18 (8.0) | 60  NR  <11 | 173  NR  NR | 302  NR  NR | 6  <11  <11 |
| **Discontinuation (variable follow-up), months** | | | | | | | | |
| Total exposures  Total exposure time  Rate (per 100 person-years)  95% CI | 36  209,298  6.26  4.52–8.68 | 4  14,267  10.21  3.83–27.19 | 6  76,918  2.84  1.28–6.32 | 18  91,902  7.13  4.49–11.32 | 8  26,211  11.11  5.56–22.22 | 18  75,390  8.69  5.48–13.79 | 17  131,549  4.7  2.92–7.57 | 1  2359  15.43  2.17–109.54 |
| **Discontinuation at fixed time periods (cumulative), *n* (%)** | | | | | | | | |
| *n*  12 months | 275  24 (8.7) | 21  <11 | 97  <11 | 119  12 (10.1) | 38  <11 | 98  12 (12.2) | 173  NR | 4  <11 |
| *n*  24 months | 44  <11 | 5  <11 | 22  <11 | 13  <11 | 4  <11 | 14  <11 | 30  <11 | 0  0 (0.0) |
| **TTD, days** | | | | | | | | |
| *n*  Mean (SD)  Median  IQR (min, max) | 36  4.5 (4.0)  2.9  1.1–5.5  (0.3, 14.6) | 4  3.8 (4.8)  1.9  0.8–6.8  (0.6, 10.8) | 6  9.0 (4.1)  9.2  4.5–12.2  (4.4, 14.6) | 18  3.4 (3.1)  2.5  1.0–5.0  (0.5, 13.3) | 8  3.8 (3.8)  2.4  1.5–6.1  (0.3, 10.0) | 18  5.4 (4.4)  4.4  1.7–9.2  (0.5, 14.6) | 17  3.6 (3.6)  2.4  1.0–4.5  (0.3, 13.3) | 1  2.8 (..)  2.8  2.8–2.8  (2.8, 2.8) |
| **Probability of TTD (Kaplan–Meier estimate)** | | | | | | | | |
| **TTD, months** | | | | | | | | |
| Censored  Events  25% survival time  95% CI  Median survival time  95% CI | 0  481  0  0.00–0.00  0  0.00–0.00 | 0  0  0  2.83, –^†^  0  0.00–0.00 | 0  166  0  0.00–0.00  0  0.00–0.00 | 0  225  0  0.00–0.00  0  0.00–0.00 | 0  60  0  9.69, –^†^  0  0.00–0.00 | 0  173  0  0.00–0.00  0  0.00–0.00 | 0  302  0  0.00–0.00  0  0.00–0.00 | 0  6  0  2.83, –^†^  0  2.83, –^†^ |
| **12 months post index**  No. fail  No. at risk  Failure function  95% CI | 3  194  0.085  0.060–0.118 | 0  14  0.162  0.063–0.383 | 1  74  0.088  0.063–0.383 | 0  0  0  0.063–0.383 | 0  0  0  0.063–0.383 | 2  69  0.12  0.074–0.191 | 0  3  0.2  0.074–0.191 | 0  0  0  0.074–0.191 |

^†^Lower and/or upper bounds are outside of the observable range (i.e., follow-up).

.. Sample too small to calculate SD.

Primary data suppression applied to all values with fewer than 11 cases (<11), with secondary suppression (NR) applied where required to protect primary suppression.

Abbreviations: CI, confidence interval; IQR, interquartile range; NA, nucleos(t)ide analog; NR, not recorded; SD, standard deviation; TAF, tenofovir alafenamide fumarate; TDF, tenofovir disoproxil fumarate; TTD, time to discontinuation.

**TABLE S3.** Sensitivity analysis in the pre-, peri-, and post-pandemic periods to assess adherence to newly initiated NA treatment, stratified by age, index NA prescriber specialty, and index NA.

| **SENSITIVITY ANALYSIS: Pre-pandemic period (for patients indexed prior to and excluding April 7, 2020)** | | | | | | | | |
| --- | --- | --- | --- | --- | --- | --- | --- | --- |
|  | **Age at index** | | | | | **Indexed NA** | | |
|  | **Overall**  **(*n* = 1558)** | **18–34 years**  **(*n* = 109)** | **35–49 years**  **(*n* = 712)** | **50–64 years**  **(*n* = 630)** | **≥65 years**  **(*n* = 107)** | **Entecavir**  **(*n* = 1035)** | **TAF**  **(*n* = 333)** | **TDF**  **(*n* = 190)** |
| **PDC (variable follow-up)** | | | | | | | | |
| *n*  Mean (SD)  Median  IQR  (min, max) | 1558  0.88 (0.24)  1.00  0.91–1.00  (0.00, 1.00) | 109  0.82 (0.28)  0.98  0.78–1.00  (0.04, 1.00) | 712  0.9 (0.22)  0.99  0.92–1.00  (0.02, 1.00) | 630  0.88 (0.24)  1.00  0.92–1.00  (0.00, 1.00) | 107  0.84 (0.29)  1.00  0.86–1.00  (0.03, 1.00) | 1035  0.86 (0.25)  0.99  0.86–1.00  (0.00, 1.00) | 333  0.94 (0.19)  1.00  0.99–1.00  (0.02, 1.00) | 190  0.9 (0.21)  0.99  0.92–1.00  (0.02, 1.00) |
| **PDC (variable follow-up), binary****, *n* (%)** | | | | | | | | |
| *n*  <80% (nonadherent)  ≥80% (adherent) | 1558  272 (17.5)  1286 (82.5) | 109  30 (27.5)  79 (72.5) | 712  104 (14.6)  608 (85.4) | 630  113 (17.9)  517 (82.1) | 107  25 (23.4)  82 (76.6) | 1035  221 (21.4)  814 (78.6) | 333  23 (6.9)  310 (93.1) | 190  28 (14.7)  162 (85.3) |
| **PDC, at 12 months** | | | | | | | | |
| *n*  Mean (SD)  Median  IQR (mix, max) | 1459  0.91 (0.21)  1.00  0.97–1.00  (0.02, 1.00) | 102  0.88 (0.24)  1.00  0.93–1.00  (0.08, 1.00) | 695  0.93 (0.19)  1.00  0.98–1.00  (0.02, 1.00) | 575  0.91 (0.22)  1.00  0.96–1.00  (0.02, 1.00) | 87  0.88 (0.27)  1.00  0.96–1.00  (0.08, 1.00) | 956  0.9 (0.22)  1.00  0.95–1.00  (0.02, 1.00) | 322  0.94 (0.18)  1.00  1.00–1.00  (0.03, 1.00) | 181  0.93 (0.19)  1.00  0.98–1.00  (0.08, 1.00) |
| **PDC, at 12 months, binary, *n* (%)** | | | | | | | | |
| *n*  <80% (nonadherent)  ≥80% (adherent) | 1459  193 (13.2)  1266 (86.8) | 102  19 (18.6)  83 (81.4) | 695  77 (11.1)  618 (88.9) | 575  83 (14.4)  492 (85.6) | 87  14 (16.1)  73 (83.9) | 956  152 (15.9)  804 (84.1) | 322  24 (7.5)  298 (92.5) | 181  17 (9.4)  164 (90.6) |
| **PDC, at 24 months** | | | | | | | | |
| *n*  Mean (SD)  Median  IQR (min, max) | 1324  0.9 (0.23)  1.00  0.93–1.00  (0.01, 1.00) | 95  0.86 (0.27)  1.00  0.88–1.00  (0.04, 1.00) | 663  0.91 (0.21)  1.00  0.96–1.00  (0.02, 1.00) | 508  0.89 (0.23)  1.00  0.92–1.00  (0.01, 1.00) | 58  0.8 (0.33)  1.00  0.59–1.00  (0.04, 1.00) | 862  0.88 (0.24)  1.00  0.89–1.00  (0.01, 1.00) | 294  0.94 (0.18)  1.00  0.99–1.00  (0.03, 1.00) | 168  0.91 (0.21)  1.00  0.95–1.00  (0.04, 1.00) |
| **PDC, at 24 months, binary, *n* (%)** | | | | | | | | |
| *n*  <80% (nonadherent)  ≥80% (adherent) | 1324  193 (14.6)  1131 (85.4) | 95  20 (21.1)  75 (78.9) | 663  79 (11.9)  584 (88.1) | 508  79 (15.6)  429 (84.4) | 58  15 (25.9)  43 (74.1) | 862  155 (18.0)  707 (82.0) | 294  19 (6.5)  275 (93.5) | 168  19 (11.3)  149 (88.7) |
| **PDC, at 36 months** | | | | | | | | |
| *n*  Mean (SD)  Median  IQR (min, max) | 1198  0.89 (0.23)  1.00  0.92–1.00  (0.01, 1.00) | 85  0.85 (0.27)  0.99  0.85–1.00  (0.04, 1.00) | 625  0.91 (0.21)  1.00  0.95–1.00  (0.02, 1.00) | 445  0.89 (0.23)  1.00  0.91–1.00  (0.01, 1.00) | 43  0.79 (0.34)  0.99  0.69–1.00  (0.03, 1.00) | 784  0.87 (0.25)  0.99  0.88–1.00  (0.01, 1.00) | 264  0.95 (0.18)  1.00  0.99–1.00  (0.03, 1.00) | 150  0.9 (0.22)  0.99  0.95–1.00  (0.03, 1.00) |
| **PDC, at 36 months, binary, *n* (%)** | | | | | | | | |
| *n*  <80% (nonadherent)  ≥80% (adherent) | 1198  185 (15.4)  1013 (84.6) | 85  18 (21.2)  67 (78.8) | 625  82 (13.1)  543 (86.9) | 445  74 (16.6)  371 (83.4) | 43  11 (25.6)  32 (74.4) | 784  151 (19.3)  633 (80.7) | 264  16 (6.1)  248 (93.9) | 150  18 (12.0)  132 (88.0) |
| **PDC, at 48 months** | | | | | | | | |
| *n*  Mean (SD)  Median  IQR  (min, max) | 1049  0.88 (0.24)  0.99  0.92–1.00  (0.01, 1.00) | 73  0.83 (0.28)  0.99  0.79–1.00  (0.04, 1.00) | 568  0.9 (0.22)  0.99  0.94–1.00  (0.02, 1.00) | 379  0.88 (0.24)  1.00  0.89–1.00  (0.01, 1.00) | 29  0.76 (0.35)  0.98  0.58–1.00  (0.03, 1.00) | 706  0.86 (0.25)  0.99  0.87–1.00  (0.01, 1.00) | 210  0.95 (0.17)  1.00  0.99–1.00  (0.03, 1.00) | 133  0.9 (0.22)  0.99  0.94–1.00  (0.02, 1.00) |
| **PDC, at 48 months, binary, *n* (%)** | | | | | | | | |
| *n*  <80% (nonadherent)  ≥80% (adherent) | 1049  178 (17.0)  871 (83.0) | 73  NR  NR | 568  81 (14.3)  487 (85.7) | 379  68 (17.9)  311 (82.1) | 29  <11  NR | 706  148 (21.0)  558 (79.0) | 210  13 (6.2)  197 (93.8) | 133  17 (12.8)  116 (87.2) |
| **PDC, at 60 months** | | | | | | | | |
| *n*  Mean (SD)  Median  IQR (min, max) | 814  0.88 (0.24)  0.99  0.90–1.00  (0.00, 1.00) | 59  0.80 (0.3)  0.98  0.65–1.00  (0.04, 1.00) | 466  0.89 (0.23)  0.99  0.92–1.00  (0.02, 1.00) | 271  0.87 (0.24)  0.99  0.91–1.00  (0.00, 1.00) | 18  0.8 (0.32)  0.98  0.66–1.00  (0.03, 1.00) | 599  0.86 (0.25)  0.98  0.87–1.00  (0.00, 1.00) | 95  0.93 (0.21)  1.00  0.99–1.00  (0.03, 1.00) | 120  0.90 (0.22)  0.99  0.95–1.00  (0.02, 1.00) |
| **PDC, at 60 months, binary, *n* (%)** | | | | | | | | |
| *n*  <80% (nonadherent)  ≥80% (adherent) | 814  145 (17.8)  669 (82.2) | 59  NR  NR | 466  72 (15.5)  394 (84.5) | 271  50 (18.5)  221 (81.5) | 18  <11  NR | 599  123 (20.5)  476 (79.5) | 95  <11  NR | 120  NR  NR |
| **SENSITIVITY ANALYSIS: Peri-pandemic period (for patients indexed from April 7, 2020, to September 30, 2021)** | | | | | | | | |
|  | **Age at index** | | | | | **Indexed NA** | | |
|  | **Overall**  **(*n* = 434)** | **18–34 years**  **(*n* = 31)** | **35–49 years**  **(*n* = 180)** | **50–64 years**  **(*n* = 184)** | **≥65 years**  **(*n* = 39)** | **Entecavir**  **(*n* = 165)** | **TAF**  **(*n* = 260)** | **TDF**  **(*n* = 9)** |
| **PDC (variable follow-up)** | | | | | | | | |
| *n*  Mean (SD)  Median  IQR (min, max) | 434  0.95 (0.15)  1.00  1.00–1.00  (0.04, 1.00) | 31  0.93 (0.19)  1.00  1.00–1.00  (0.23, 1.00) | 180  0.95 (0.16)  1.00  1.00–1.00  (0.04, 1.00) | 184  0.97 (0.1)  1.00  1.00–1.00  (0.07, 1.00) | 39  0.92 (0.22)  1.00  0.99–1.00  (0.05, 1.00) | 165  0.93 (0.18)  1.00  0.98–1.00  (0.04, 1.00) | 260  0.98 (0.11)  1.00  1.00–1.00  (0.17, 1.00) | 9  0.72 (0.34)  0.87  0.51–1.00  (0.05, 1.00) |
| **PDC (variable follow-up), binary, *n* (%)** | | | | | | | | |
| *n*  <80% (nonadherent)  ≥80% (adherent) | 434  32 (7.4)  402 (92.6) | 31  <11  NR | 180  15 (8.3)  165 (91.7) | 184  <11  NR | 39  <11  NR | 165  20 (12.1)  145 (87.9) | 260  <11  NR | 9  <11  <11 |
| **PDC, at 12 months** | | | | | | | | |
| *n*  Mean (SD)  Median  IQR  (min, max) | 392  0.96 (0.14)  1.00  1.00–1.00  (0.05, 1.00) | 28  0.96 (0.12)  1.00  1.00–1.00  (0.50, 1.00) | 171  0.95 (0.15)  1.00  1.00–1.00  (0.05, 1.00) | 162  0.97 (0.11)  1.00  1.00–1.00  (0.07, 1.00) | 31  0.94 (0.19)  1.00  0.99–1.00  (0.08, 1.00) | 149  0.95 (0.16)  1.00  0.99–1.00  (0.05, 1.00) | 234  0.98 (0.1)  1.00  1.00–1.00  (0.17, 1.00) | 9  0.74 (0.33)  0.87  0.64–1.00  (0.08, 1.00) |
| **PDC, at 12 months, binary, *n* (%)** | | | | | | | | |
| *n*  <80% (nonadherent)  ≥80% (adherent) | 392  26 (6.6)  366 (93.4) | 28  <11  NR | 171  13 (7.6)  158 (92.4) | 162  <11  NR | 31  <11  NR | 149  15 (10.1)  134 (89.9) | 234  <11  NR | 9  <11  <11 |
| **PDC, at 24 months** | | | | | | | | |
| *n*  Mean (SD)  Median  IQR (min, max) | 349  0.96 (0.13)  1.00  1.00–1.00  (0.05, 1.00) | 19  0.97 (0.11)  1.00  1.00–1.00  (0.51, 1.00) | 159  0.96 (0.14)  1.00  1.00–1.00  (0.17, 1.00) | 148  0.97 (0.11)  1.00  1.00–1.00  (0.07, 1.00) | 23  0.92 (0.23)  1.00  0.99–1.00  (0.05, 1.00) | 134  0.95 (0.15)  1.00  0.99–1.00  (0.07, 1.00) | 208  0.98 (0.1)  1.00  1.00–1.00  (0.17, 1.00) | 7  0.71 (0.39)  1.00  0.40–1.00  (0.05, 1.00) |
| **PDC, at 24 months, binary, *n* (%)** | | | | | | | | |
| *n*  <80% (nonadherent)  ≥80% (adherent) | 349  22 (6.3)  327 (93.7) | 19  <11  NR | 159  11 (6.9)  148 (93.1) | 148  <11  NR | 23  <11  NR | 134  14 (10.4)  120 (89.6) | 208  <11  NR | 7  <11  <11 |
| **PDC, at 36 months** | | | | | | | | |
| n  Mean (SD)  Median  IQR (min, max) | 149  0.94 (0.17)  1.00  0.99–1.00  (0.05, 1.00) | 8  0.93 (0.17)  1.00  0.98–1.00  (0.51, 1.00) | 68  0.94 (0.17)  1.00  0.99–1.00  (0.18, 1.00) | 64  0.94 (0.16)  1.00  0.98–1.00  (0.07, 1.00) | 9  0.84 (0.32)  0.99  0.97–1.00  (0.05, 1.00) | 57  0.9 (0.2)  1.00  0.96–1.00  (0.07, 1.00) | 87  0.98 (0.09)  1.00  1.00–1.00  (0.18, 1.00) | 5  0.59 (0.41)  0.51  0.40–1.00  (0.05, 1.00) |
| **PDC, at 36 months, binary, *n* (%)** | | | | | | | | |
| *n*  <80% (nonadherent)  ≥80% (adherent) | 149  14 (9.4)  135 (90.6) | 8  <11  <11 | 68  <11  NR | 64  <11  NR | 9  <11  <11 | 57  <11  NR | 87  <11  NR | 5  <11  <11 |
| **SENSITIVITY ANALYSIS: Post-pandemic period (for patients indexed from October 1, 2021, to August 31, 2023)** | | | | | | | | |
|  | **Age at index** | | | | | **Indexed NA** | | |
|  | **Overall**  **(*n* = 481)** | **18–34 years**  **(*n* = 30)** | **35–49 years**  **(*n* = 166)** | **50–64 years**  **(*n* = 225)** | **≥65 years**  **(*n* = 60)** | **Entecavir**  **(*n* = 173)** | **TAF**  **(*n* = 302)** | **TDF**  **(*n* = 6)** |
| **PDC (variable follow-up)** | | | | | | | | |
| *n*  Mean (SD)  Median  IQR (min, max) | 481  0.92 (0.2)  1.00  0.97–1.00  (0.02, 1.00) | 30  0.88 (0.28)  1.00  0.93–1.00  (0.03, 1.00) | 166  0.95 (0.13)  1.00  0.97–1.00  (0.14, 1.00) | 225  0.92 (0.21)  1.00  0.98–1.00  (0.02, 1.00) | 60  0.88 (0.26)  1.00  0.94–1.00  (0.05, 1.00) | 173  0.88 (0.24)  1.00  0.91–1.00  (0.03, 1.00) | 302  0.95 (0.17)  1.00  0.99–1.00  (0.02, 1.00) | 6  0.88 (0.27)  0.99  0.97–1.00  (0.33, 1.00) |
| **PDC (variable follow-up), binary, *n* (%)** | | | | | | | | |
| *n*  <80% (nonadherent)  ≥80% (adherent) | 481  53 (11.0)  428 (89.0) | 30  <11  NR | 166  12 (7.2)  154 (92.8) | 225  26 (11.6)  199 (88.4) | 60  NR  NR | 173  32 (18.5)  141 (81.5) | 302  NR  NR | 6  <11  <11 |
| **PDC, at 12 months** | | | | | | | | |
| *n*  Mean (SD)  Median  IQR (min, max) | 275  0.93 (0.19)  1.00  0.03–1.00  (0.97, 1.00) | 21  0.85 (0.3)  1.00  0.05–1.00  (0.91, 1.00) | 97  0.96 (0.11)  1.00  0.23–1.00  (0.97, 1.00) | 119  0.92 (0.22)  1.00  0.03–1.00  (0.98, 1.00) | 38  0.91 (0.2)  1.00  0.17–1.00  (0.96, 1.00) | 98  0.9 (0.22)  1.00  0.05–1.00  (0.92, 1.00) | 173  0.94 (0.17)  1.00  0.03–1.00  (0.98, 1.00) | 4  0.82 (0.33)  0.99  0.33–1.00  (0.65, 1.00) |
| **PDC, at 12 months, binary, *n* (%)** | | | | | | | | |
| *n*  <80% (nonadherent)  ≥80% (adherent) | 275  27 (9.8)  248 (90.2) | 21  <11  NR | 97  <11  NR | 119  15 (12.6)  104 (87.4) | 38  <11  NR | 98  NR  NR | 173  13 (7.5)  160 (92.5) | 4  <11  <11 |
| **PDC, at 24 months** | | | | | | | | |
| *n*  Mean (SD)  Median  IQR (mix, max) | 44  0.89 (0.26)  1.00  0.96–1.00  (0.03, 1.00) | 5  0.68 (0.46)  1.00  0.37–1.00  (0.03, 1.00) | 22  0.95 (0.11)  1.00  0.94–1.00  (0.56, 1.00) | 13  0.92 (0.26)  1.00  0.99–1.00  (0.07, 1.00) | 4  0.77 (0.45)  1.00  0.55–1.00  (0.09, 1.00) | 14  0.82 (0.35)  1.00  0.77–1.00  (0.03, 1.00) | 30  0.93 (0.2)  1.00  0.98–1.00  (0.09, 1.00) | 0  0 (0)  0  0.00–0.00  (0.00, 0.00) |
| **PDC, at 24 months, binary, *n* (%)** | | | | | | | | |
| *n*  <80% (nonadherent)  ≥80% (adherent) | 44  <11  NR | 5  <11  <11 | 22  <11  NR | 13  <11  NR | 4  <11  <11 | 14  <11  <11 | 30  <11  NR | 0  0 (0.0)  0 (0.0) |

Primary data suppression applied to all values with fewer than 11 cases (<11), with secondary suppression (NR) applied where required to protect primary suppression.

Abbreviations: IQR, interquartile range; NA, nucleos(t)ide analog; NR, not recorded; PDC, proportion of days covered; SD, standard deviation; TAF, tenofovir alafenamide fumarate; TDF, tenofovir disoproxil fumarate.

## Sensitivity analysis of NA treatment adherence

In the pre-pandemic period, there were limited numerical differences overall and across stratifications compared with the main analysis. The proportion of patients adherent to NA treatment was 82.5% (*n* = 1286) and the overall mean (SD) PDC over variable follow-up was 0.88 (0.24). Higher proportions of patients adhered to their index NA treatment in the peri- (*n* = 402; 92.6%) and post-pandemic (*n* = 428, *n* = 89.0%) periods, compared with the main analysis. Mean (SD) PDC values in the peri- and post-pandemic periods were 0.95 (0.15) and 0.92 (0.20), respectively.
